# Factorial validity and comparability of the six translations of the Rivermead Post-Concussion Symptoms Questionnaire translations: results from the CENTER-TBI study

**DOI:** 10.1186/s41687-023-00632-5

**Published:** 2023-09-08

**Authors:** Marina Zeldovich, Fabian Bockhop, Amra Covic, Isabelle Mueller, Suzanne Polinder, Ana Mikolic, Marjolein van der Vlegel, Nicole von Steinbuechel, CENTER-TBI participants and investigators, Cecilia Åkerlund, Krisztina Amrein, Nada Andelic, Lasse Andreassen, Audny Anke, Anna Antoni, Gérard Audibert, Philippe Azouvi, Maria Luisa Azzolini, Ronald Bartels, Pál Barzó, Romuald Beauvais, Ronny Beer, Bo-Michael Bellander, Antonio Belli, Habib Benali, Maurizio Berardino, Luigi Beretta, Morten Blaabjerg, Peter Bragge, Alexandra Brazinova, Vibeke Brinck, Joanne Brooker, Camilla Brorsson, Andras Buki, Monika Bullinger, Manuel Cabeleira, Alessio Caccioppola, Emiliana Calappi, Maria Rosa Calvi, Peter Cameron, Guillermo Carbayo Lozano, Marco Carbonara, Simona Cavallo, Giorgio Chevallard, Arturo Chieregato, Giuseppe Citerio, Hans Clusmann, Mark Coburn, Jonathan Coles, Jamie D. Cooper, Marta Correia, Amra Čović, Nicola Curry, Endre Czeiter, Marek Czosnyka, Claire Dahyot-Fizelier, Paul Dark, Helen Dawes, Véronique De Keyser, Vincent Degos, Francesco Della Corte, Hugo den Boogert, Bart Depreitere, Đula Đilvesi, Abhishek Dixit, Emma Donoghue, Jens Dreier, Guy-Loup Dulière, Ari Ercole, Patrick Esser, Erzsébet Ezer, Martin Fabricius, Valery L. Feigin, Kelly Foks, Shirin Frisvold, Alex Furmanov, Pablo Gagliardo, Damien Galanaud, Dashiell Gantner, Guoyi Gao, Pradeep George, Alexandre Ghuysen, Lelde Giga, Ben Glocker, Jagoš Golubovic, Pedro A. Gomez, Johannes Gratz, Benjamin Gravesteijn, Francesca Grossi, Russell L. Gruen, Deepak Gupta, Juanita A. Haagsma, Iain Haitsma, Raimund Helbok, Eirik Helseth, Lindsay Horton, Jilske Huijben, Peter J. Hutchinson, Bram Jacobs, Stefan Jankowski, Mike Jarrett, Ji-yao Jiang, Faye Johnson, Kelly Jones, Mladen Karan, Angelos G. Kolias, Erwin Kompanje, Daniel Kondziella, Evgenios Kornaropoulos, Lars-Owe Koskinen, Noémi Kovács, Ana Kowark, Alfonso Lagares, Linda Lanyon, Steven Laureys, Fiona Lecky, Didier Ledoux, Rolf Lefering, Valerie Legrand, Aurelie Lejeune, Leon Levi, Roger Lightfoot, Hester Lingsma, Andrew I. R. Maas, Ana M. Castaño-León, Marc Maegele, Marek Majdan, Alex Manara, Geoffrey Manley, Costanza Martino, Hugues Maréchal, Julia Mattern, Catherine McMahon, Béla Melegh, David Menon, Tomas Menovsky, Ana Mikolic, Benoit Misset, Visakh Muraleedharan, Lynnette Murray, Ancuta Negru, David Nelson, Virginia Newcombe, Daan Nieboer, József Nyirádi, Otesile Olubukola, Matej Oresic, Fabrizio Ortolano, Aarno Palotie, Paul M. Parizel, Jean-François Payen, Natascha Perera, Vincent Perlbarg, Paolo Persona, Wilco Peul, Anna Piippo-Karjalainen, Matti Pirinen, Dana Pisica, Horia Ples, Suzanne Polinder, Inigo Pomposo, Jussi P. Posti, Louis Puybasset, Andreea Radoi, Arminas Ragauskas, Rahul Raj, Malinka Rambadagalla, Isabel Retel Helmrich, Jonathan Rhodes, Sylvia Richardson, Sophie Richter, Samuli Ripatti, Saulius Rocka, Cecilie Roe, Olav Roise, Jonathan Rosand, Jeffrey V. Rosenfeld, Christina Rosenlund, Guy Rosenthal, Rolf Rossaint, Sandra Rossi, Daniel Rueckert, Martin Rusnák, Juan Sahuquillo, Oliver Sakowitz, Renan Sanchez-Porras, Janos Sandor, Nadine Schäfer, Silke Schmidt, Herbert Schoechl, Guus Schoonman, Rico Frederik Schou, Elisabeth Schwendenwein, Charlie Sewalt, Ranjit D. Singh, Toril Skandsen, Peter Smielewski, Abayomi Sorinola, Emmanuel Stamatakis, Simon Stanworth, Robert Stevens, William Stewart, Ewout W. Steyerberg, Nino Stocchetti, Nina Sundström, Riikka Takala, Viktória Tamás, Tomas Tamosuitis, Mark Steven Taylor, Braden Te Ao, Olli Tenovuo, Alice Theadom, Matt Thomas, Dick Tibboel, Marjolein Timmers, Christos Tolias, Tony Trapani, Cristina Maria Tudora, Andreas Unterberg, Peter Vajkoczy, Shirley Vallance, Egils Valeinis, Zoltán Vámos, Mathieu van der Jagt, Gregory Van der Steen, Joukje van der Naalt, Jeroen T. J. M. van Dijck, Inge A. M. van Erp, Thomas A. van Essen, Wim Van Hecke, Caroline van Heugten, Dominique Van Praag, Ernest van Veen, Thijs Vande Vyvere, Roel P. J. van Wijk, Alessia Vargiolu, Emmanuel Vega, Kimberley Velt, Jan Verheyden, Paul M. Vespa, Anne Vik, Rimantas Vilcinis, Victor Volovici, Nicole von Steinbüchel, Daphne Voormolen, Petar Vulekovic, Kevin K. W. Wang, Daniel Whitehouse, Eveline Wiegers, Guy Williams, Lindsay Wilson, Stefan Winzeck, Stefan Wolf, Zhihui Yang, Peter Ylén, Alexander Younsi, Frederick A. Zeiler, Veronika Zelinkova, Agate Ziverte, Tommaso Zoerle

**Affiliations:** 1 Institute of Medical Psychology and Medical Sociology, University Medical Center Göttingen, Göttingen, Germany; 2 Department of Psychiatry, Columbia University Medical Center, New York, NY USA; 3 Department of Public Health, Erasmus MC, University Medical Center Rotterdam, Rotterdam, The Netherlands; 4 Department of Psychology, The University of British Columbia, Vancouver, BC Canada; 5 Rehabilitation Research Program, Centre for Aging SMART at Vancouver Coastal Health, Vancouver, BC Canada

**Keywords:** Traumatic brain injury, Post-concussion symptoms, Measurement invariance, Rivermead Post-Concussion Symptoms Questionnaire

## Abstract

**Background:**

Comparison of patient-reported outcomes in multilingual studies requires evidence of the equivalence of translated versions of the questionnaires. The present study examines the factorial validity and comparability of six language versions of the Rivermead Post-Concussion Symptoms Questionnaire (RPQ) administered to individuals following traumatic brain injury (TBI) in the Collaborative European NeuroTrauma Effectiveness Research (CENTER-TBI) study.

**Methods:**

Six competing RPQ models were estimated using data from Dutch (n = 597), English (n = 223), Finnish (n = 213), Italian (n = 268), Norwegian (n = 263), and Spanish (n = 254) language samples recruited six months after injury. To determine whether the same latent construct was measured by the best-fitting model across languages and TBI severity groups (mild/moderate vs. severe), measurement invariance (MI) was tested using a confirmatory factor analysis framework.

**Results:**

The results did not indicate a violation of the MI assumption. The six RPQ translations were largely comparable across languages and were able to capture the same construct across TBI severity groups. The three-factor solution comprising emotional, cognitive, and somatic factors provided the best fit with the following indices for the total sample: χ^2^ (101) = 647.04, ${\chi }^{2}/df$= 6.41, *p* < 0.001, CFI = 0.995, TLI = 0.994, RMSEA = 0.055, CI_90%_[0.051, 0.059], SRMR = 0.051.

**Conclusion:**

The RPQ can be used in international research and clinical settings, allowing direct comparisons of scores across languages analyzed within the full spectrum of TBI severity. To strengthen the aggregated applicability across languages, further analyses of the utility of the response scale and comparisons between different translations of the RPQ at the item level are recommended.

**Supplementary Information:**

The online version contains supplementary material available at 10.1186/s41687-023-00632-5.

## Background

Traumatic brain injury (TBI) is a condition characterized by changes in brain functioning caused by external head trauma [1]. It imposes life-long limitations [2] and leads to a range of physical, emotional, and cognitive disabilities, impacting functioning of affected individuals [1]. The burden of TBI extends to family caregivers [3], as well as health and economic systems [2].

Individuals after TBI can especially suffer from a range of post-concussion symptoms (PCS), which may persist much longer than initially expected [4]. These symptoms encompass physical (e.g., headaches or nausea), cognitive (e.g., a diminished ability to concentrate), and emotional/behavioral (e.g., depressiveness or fatigue) impairments [5]. PCS are commonly reported after mild to moderate TBI [6], but individuals following severe TBI also experience similar deficits [7, 8], referred to as PC-like symptoms.

To assess PCS, researchers and clinicians often rely on the subjective experiences of those affected using patient-reported outcome measures (PROMs), such as the Rivermead Post-Concussion Symptoms Questionnaire (RPQ) [6]. The RPQ is widely used to assess self-reported PCS. For the Collaborative European NeuroTrauma Effectiveness Research study (CENTER-TBI; clinicaltrials.gov NCT02210221), which was designed to examine treatment and outcomes of individuals after TBI in 18 countries [9], translations and linguistic validations were performed for the RPQ, resulting in eleven additional versions [10].

In multilingual studies, the equivalence of translated PROMs, in terms of their conceptual alignment with the original version and other translations, cultural relevance, acceptability to the target populations, and psychometrical comparability, is essential for language and country comparisons, as well as data aggregation in multilingual studies [11]. Measurement invariance (MI) analysis is a valuable tool to determine whether the translations of an instrument measure the same construct [12]. In particular, MI analysis investigates if the differences in observed variables across language versions are solely attributed to differences in latent means.

Therefore, the main aim of the present study is to provide empirical evidence of MI for the RPQ in six European languages. The RPQ has been declared a unidimensional PROM, but this property could not be replicated across translations, including English-speaking samples e.g., [13]. Thus, the factorial structure of the RPQ is examined to find the best-fitting model prior to MI analyses. In addition, the study seeks to explore whether the same construct is measured across the spectrum of TBI severity.

Once the assumption of MI is met, differences in RPQ scores between language samples will be due to true differences in self-reported PCS and not to differences in translation allowing for data aggregation and direct comparisons.

## Methods

### Study design and participants

Data were collected from December 2014 to December 2019 within the CENTER-TBI study involving 63 centers in 18 countries in Europe and Israel. A total of 4,509 individuals after TBI were enrolled in the core study. Inclusion criteria for study participation were clinical diagnosis of TBI and indication for computed tomography (CT) scan, enrollment within 24 h after injury, and informed consent for study participation. Written informed consent was obtained according to the local and national legislations for all patients (either by the patients or the legal representatives) and documented in the electronic case report form. To avoid bias in outcome assessment, patients with severe pre-existing neurological disorders were excluded from the study. Individuals were either seen in the emergency room (ER) and then discharged or either admitted to the hospital ward or intensive care unit (ICU). More detailed description of the study design is provided by Steyerberg et al. [14]. Data were retrieved from the CENTER-TBI database via Neurobot tool (core data set 2.1, November 2019).

**Fig. 1:**
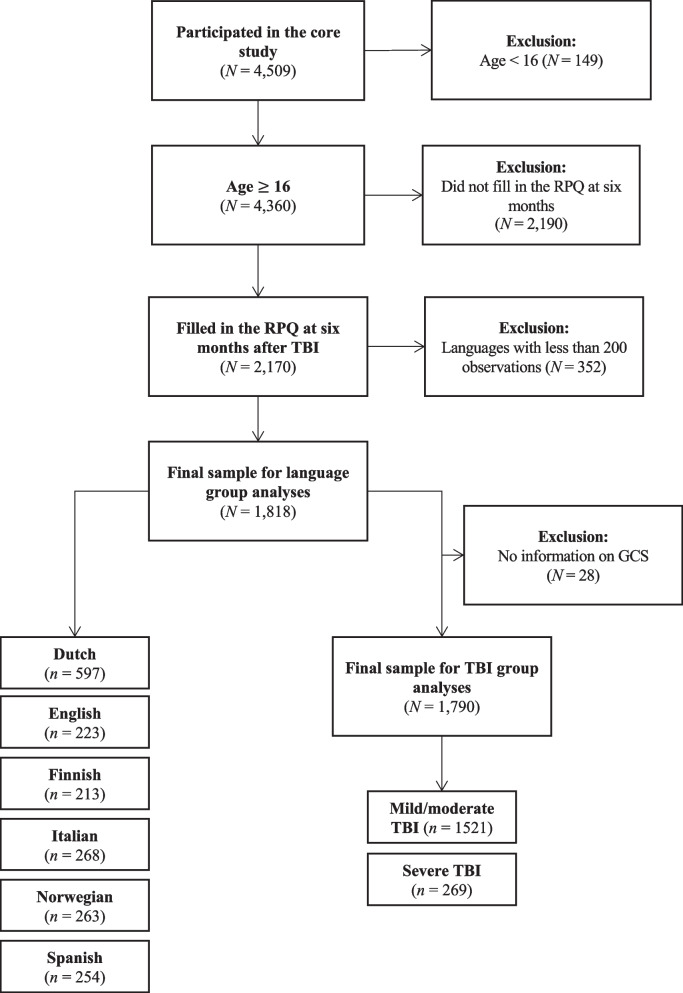
Sample attrition

The following analyses were limited to individuals aged 16 years of age or older who completed the RPQ six months after TBI. Following the recommendation for MI analyses, we included language samples with at least 200 participants (N = 1,818). Additional analyses on TBI severity involved individuals with available information on the Glasgow Coma Scale (GCS) [15] score at baseline (N = 1,790). For the composition of the study sample, see Fig. 1.

### Sample characteristics

Sociodemographic characteristics were collected at study enrollment and included sex, age, education (in groups and years), employment status, marital status, and living situation. Language samples were identified according to the languages spoken in the participating sites. For more details on language sample compositions in the CENTER-TBI study, see von Steinbuechel et al. [13].

The following variables were used to characterize premorbid and injury-related factors: mental health status before the injury (presence vs. absence of prior psychiatric diseases), cause of injury, clinical care pathways (ER, ward, ICU), and TBI severity measured using the Glasgow Coma Scale at baseline (GCS) [15] combined with information on abnormalities on the CT scan (uncomplicated mild, complicated mild, moderate, and severe TBI) [15, 16]. The functional recovery status at six months was rated using the Glasgow Outcome Scale – Extended (GOSE) [17]. The total injury severity score (ISS) and the brain injury severity score from the Abbreviated Injury Scale (AIS) [18] assessed total injury severity and brain injury severity, respectively.

### The Rivermead Post-Concussion Symptoms Questionnaire

The Rivermead Post-Concussion Symptoms Questionnaire (RPQ) [6] assesses 16 symptoms including headaches, dizziness, nausea and/or vomiting, noise sensitivity, sleep disturbance, fatigue, irritability, depression, frustration, forgetfulness and poor memory, poor concentration, slow thinking, blurred vision, light sensitivity, double vision, and restlessness. Individuals are asked to rate the symptoms over the last 24 h compared with their condition before the TBI using a five-point Likert-type scale (from 0 “not experienced at all” to 4 “a severe problem”). Based on the originally proposed unidimensional factor structure, the total score ranges from 0 to 64 with higher values indicating greater impairment, whereby values of “1” indicating no more of a problem than before TBI are treated as zero. For clinical screening, a cut-off score of 12 can be applied [19]. The factorial structure of the RPQ has so far been the subject of several studies [19–24] and no agreement on an ultimate solution has yet been reached. Initial analyses of the unidimensional structure using data collected in the CENTER-TBI study also revealed rather poor model fit across language samples [13].

### Statistical analyses

#### Descriptive statistics and language-samples comparisons

Prior to statistical analyses, language samples were compared by sample characteristics (esp. injury-related factors) using Kruskal–Wallis tests and pairwise U-tests to ensure their general comparability. For the pairwise U-tests, Vargha and Delaney’s effect size [25] was calculated based on the following cut-offs: groups equality (0.50), small (0.35–0.44 or 0.56–0.63), medium (0.30–0.34 or 0.64–0.70), and large effect (beyond 0.29 or 0.71). Distribution of the TBI severity groups in the language samples has been investigated applying a two-dimensional chi-squared test and computing Cramer’s V to determine the effect size. For this purpose, we first used the initial distribution containing four TBI severity groups (i.e., uncomplicated mild, complicated mild, moderate, and severe TBI), and then a collapsed classification (i.e., mild/moderate and severe TBI). The effect size was determined using Cohen’s taxonomy [26] with values of 0.10, 0.30, and 0.50 representing small, medium, and large effects, respectively. Furthermore, the distribution of PCS in language samples has been investigated.

#### Analyses of dimensionality

First, we analyzed the response pattern of the RPQ items within the language samples. The factorial structure of the RPQ translations was then examined separately for each language version using confirmatory factor analysis (CFA) with robust weighted least squares estimator (WLSMV) [27] for ordered categorical data. In the absence of agreement on the factorial structure of the RPQ, six competing models derived from previous research were estimated: the initial one-factor model [6], two two-factor models [19, 20], two three-factor models [21, 22], the first of which [21] was based on research findings presented by Gerber and Schraa [28], and one four-factor [23] model, corresponding to the study findings by Lannsjö and colleagues [24]. For more details on RPQ models estimated in the present study, see Table 1.

**Table 1:** Factorial structures of the RPQ investigated in the present study

Item label (shortened version)	One-factor model [6]	Two-factor model [19]	Two-factor model [20]	Three-factor model [21]	Three-factor model [22]	Four-factor model [23]
Headaches	RPQ	Emotional-somatic	RPQ-3	Somatic	General somatic	Vertigo
Dizziness	RPQ	Emotional-somatic	RPQ-3	Somatic	General somatic	Vertigo
Nausea	RPQ	Emotional-somatic	RPQ-3	Somatic	General somatic	Vertigo
Noise sensitivity	RPQ	Emotional-somatic	RPQ-13	Somatic	General somatic	Vertigo
Sleep disturbance	RPQ	Emotional-somatic	RPQ-13	Somatic	General somatic	Mood/somatic
Fatigue	RPQ	Emotional-somatic	RPQ-13	Somatic	Mood/cognition	Mood/somatic
Irritable	RPQ	Emotional-somatic	RPQ-13	Emotional	Mood/cognition	Mood/somatic
Depressed	RPQ	Emotional-somatic	RPQ-13	Emotional	Mood/cognition	Mood/somatic
Frustrated	RPQ	Emotional-somatic	RPQ-13	Emotional	Mood/cognition	Mood/somatic
Forgetful	RPQ	Cognitive	RPQ-13	Cognitive	Mood/cognition	Cognitive
Poor concentration	RPQ	Cognitive	RPQ-13	Cognitive	Mood/cognition	Cognitive
Longer to think	RPQ	Cognitive	RPQ-13	Cognitive	Mood/cognition	Cognitive
Blurred vision	RPQ	Emotional-somatic	RPQ-13	Somatic	Visual somatic	Vision
Light sensitivity	RPQ	Emotional-somatic	RPQ-13	Somatic	Visual somatic	Vision
Double vision	RPQ	–^a^	RPQ-13	Somatic	Visual somatic	Vision
Restless	RPQ	Emotional-somatic	RPQ-13	Emotional	General somatic	Mood/Somatic

^a^Item Double vision was excluded from the model

The fit of all estimated models was assessed by several goodness of fit indices: ${\chi }^{2}$ and degrees of freedom (*df*), as well as the ratio ${\chi }^{2}/df$, the comparative fit index (CFI) [29], the Tucker-Lewis index (TLI) [30] the root mean square error of approximation (RMSEA) [31] including 90% confidence interval (CI_90%_), and the standardized root mean square residual (SRMR). A ratio ${\chi }^{2}/df$ ≤ 2 indicate good model fit [32], CFI and TLI values larger than 0.95 indicate a good fit [33], the RMSEA values less than 0.05 signal a close fit, values from 0.05 to 0.08 a fair fit, between 0.08 and 0.10 a mediocre and above 0.10 a poor fit [34]; the same criteria apply to the CIs. SRMR values less than 0.08 demonstrate a good model fit [33]. Since the cut-off values for the CFI, TLI, and RMSEA have not yet been validated for ordinal data, interpretation should be carried out with caution [35]. Therefore, all fit measures were considered simultaneously to identify the best fitting model.

For the CFI analyses, we first used the raw data to obtain the model fit and then the data with modified items due to missing responses in some categories. Thus, responses 3 “moderate problem” or 4 “severe problem” of the items *Nausea* and *Double vision* were collapsed with the category 2 “mild problem” implying a trichotomized response format when considering 1 “no more of a problem than before TBI” as “1” and a dichotomized scale when treating “1” as “0”. For all other items, the original responses were kept to maximally retain information.

#### Measurement invariance (MI)

The best-fitting factor solution served as the basis for MI analyses across languages and for TBI severity MI analyses. Because the absence of responses in some categories would not allow for invariance testing across groups, only modified items were considered. We conducted a multi-group CFA with stepwise increasing constraints following the framework proposed by Wu and Estabrook [36] and updated by Svetina et al. [37] to make it more suitable for Likert-type scales. This approach slightly differs from the classical MI procedure. First, the baseline model testing for configural invariance was fitted. Then, this model was restrained by requiring invariance of thresholds, and thresholds and loadings across the groups. The models were stepwise compared by calculating the chi-square difference test and changes in CFI (ΔCFI) and RMSEA (ΔRMSEA). Models showing non-significant differences (p ≥ 0.05), ΔCFI < 0.01 [38], and ΔRMSEA ≤ 0.01, which is recommended for groups with unequal sample sizes [39], were considered equivalent. If the difference tests between the models were not significant, the assumption of MI was considered justified. Once the MI assumption was fulfilled, a multi-group CFA approach was again used to examine the differences between mild and moderate/severe TBI across all RPQ translations. Finally, the best-fitting model was estimated and visualized for the total study sample.

According to the original scoring [6], “1” responses indicating that a symptom posed no more of a problem than before TBI should be treated as “0”. Because participants explicitly used a five-point Likert-type scale when completing the questionnaire, “1” responses were considered in both CFA and MI analyses. However, we additionally replicated the analyses using the simplified response scale (i.e., treating 1 as 0) to achieve greater congruence with the original scoring procedure. These results are reported in the Additional file 1.

All analyses were carried out with R version 4.0.0. [40] and packages "Table 1" [41] for descriptive analyses and "lavaan" [42] for the CFA and MI testing as well as package "lavaanPlot" for model visualization [43]. The significance level was set at 5% except for pairwise comparisons, for which the Bonferroni correction was performed to avoid alpha inflation $\left( {\upalpha_{{{\text{adj}}}} = \frac{.05}{6} = 0.008} \right)$.

## Results

### Sample characteristics

The total sample comprised 1,818 participants (65.4% male) with a mean age of 49.5 ± 19.5 years (*Mdn* = 51, range 16–95) who completed the RPQ at six months after injury. Most individuals sustained a mild TBI (73.1%). The cause of injury was most commonly either a road traffic accident (41.7%) or incidental fall (43.7%). At six months after TBI, more than half of the individuals showed good recovery (GOSE: 7–8) across all language samples. For more details, see Table 2 and Additional file 1: Table S1 – Additional characteristics of the language samples.

**Table 2:** Characteristics of the language samples

Variable	Group/values	Dutch (N = 597)	English (N = 223)	Finnish (N = 213)	Italian (N = 268)	Norwegian (N = 263)	Spanish (N = 254)	Total (N = 1818)
Sex^a^	Female	229 (38.4%)	74 (33.2%)	84 (39.4%)	84 (31.3%)	83 (31.6%)	75 (29.5%)	629 (34.6%)
	Male	368 (61.6%)	149 (66.8%)	129 (60.6%)	184 (68.7%)	180 (68.4%)	179 (70.5%)	1189 (65.4%)
Age﻿	*M* (*SD*)	52.9 (19.1)	48.3 (17.1)	47.7 (19.6)	50.2 (20.6)	45.8 (19.9)	47.1 (19.4)	49.5 (19.5)
	*Mdn* [*Min*, *Max*]	57.0 [16.0, 95.0]	51.0 [16.0, 85.0]	50.0 [16.0, 89.0]	53.0 [16.0, 93.0]	48.0 [16.0, 89.0]	44.0 [16.0, 93.0]	51.0 [16.0, 95.0]
Years of education	*M* (*SD*)	14.4 (3.87)	14.9 (3.73)	13.3 (3.16)	12.4 (4.37)	14.1 (3.21)	14.7 (5.60)	14.0 (4.13)
	*Mdn* [*Min*, *Max*]	15.0 [4.00, 30.0]	14.0 [7.00, 25.0]	12.0 [9.00, 30.0]	13.0 [4.00, 25.0]	14.0 [4.00, 25.0]	15.0 [2.00, 30.0]	14.0 [2.00, 30.0]
	Missing	128 (21.4%)	30 (13.5%)	66 (31.0%)	50 (18.7%)	15 (5.7%)	45 (17.7%)	334 (18.4%)
Injury cause^a^	Incidental fall	287 (48.1%)	88 (39.5%)	98 (46.0%)	95 (35.4%)	111 (42.2%)	116 (45.7%)	795 (43.7%)
	Road traffic accident	238 (39.9%)	104 (46.6%)	70 (32.9%)	135 (50.4%)	105 (39.9%)	107 (42.1%)	759 (41.7%)
	Other	67 (11.2%)	28 (12.6%)	37 (17.4%)	31 (11.6%)	43 (16.3%)	25 (9.8%)	231 (12.7%)
	Missing	5 (0.8%)	3 (1.3%)	8 (3.8%)	7 (2.6%)	4 (1.5%)	6 (2.4%)	33 (1.8%)
Clinical pathways^a^	ER	102 (17.1%)	62 (27.8%)	51 (23.9%)	67 (25.0%)	66 (25.1%)	71 (28.0%)	419 (23.0%)
	Ward	308 (51.6%)	74 (33.2%)	99 (46.5%)	57 (21.3%)	114 (43.3%)	54 (21.3%)	706 (38.8%)
	ICU	187 (31.3%)	87 (39.0%)	63 (29.6%)	144 (53.7%)	83 (31.6%)	129 (50.8%)	693 (38.1%)
GOSE score	3	14 (2.3%)	13 (5.8%)	9 (4.2%)	24 (9.0%)	9 (3.4%)	12 (4.7%)	81 (4.5%)
	4	30 (5.0%)	18 (8.1%)	7 (3.3%)	19 (7.1%)	4 (1.5%)	13 (5.1%)	91 (5.0%)
	5	63 (10.6%)	36 (16.1%)	17 (8.0%)	27 (10.1%)	32 (12.2%)	32 (12.6%)	207 (11.4%)
	6	89 (14.9%)	34 (15.2%)	23 (10.8%)	43 (16.0%)	55 (20.9%)	22 (8.7%)	266 (14.6%)
	7	180 (30.2%)	37 (16.6%)	45 (21.1%)	45 (16.8%)	62 (23.6%)	78 (30.7%)	447 (24.6%)
	8	221 (37.0%)	84 (37.7%)	112 (52.6%)	110 (41.0%)	101 (38.4%)	97 (38.2%)	725 (39.9%)
	Missing	0 (0%)	1 (0.4%)	0 (0%)	0 (0%)	0 (0%)	0 (0%)	1 (0.1%)
TBI severity^a^	Uncomplicated mild	275 (46.1%)	83 (37.2%)	96 (45.1%)	75 (28.0%)	110 (41.8%)	80 (31.5%)	719 (39.5%)
	Complicated mild	190 (31.8%)	65 (29.1%)	74 (34.7%)	90 (33.6%)	85 (32.3%)	106 (41.7%)	610 (33.6%)
	Moderate	45 (7.5%)	11 (4.9%)	16 (7.5%)	33 (12.3%)	17 (6.5%)	12 (4.7%)	134 (7.4%)
	Severe	63 (10.6%)	51 (22.9%)	15 (7.0%)	61 (22.8%)	27 (10.3%)	52 (20.5%)	269 (14.8%)
	Missing	24 (4.0%)	13 (5.8%)	12 (5.6%)	9 (3.4%)	24 (9.1%)	4 (1.6%)	86 (4.7%)
GCS score	Mean (SD)	13.4 (3.09)	12.2 (4.38)	13.7 (2.73)	11.9 (4.13)	13.4 (3.13)	12.4 (4.51)	12.9 (3.68)
	Median [Min, Max]	15.0 [3.00, 15.0]	15.0 [3.00, 15.0]	15.0 [3.00, 15.0]	14.0 [3.00, 15.0]	15.0 [3.00, 15.0]	15.0 [3.00, 15.0]	15.0 [3.00, 15.0]
	Missing	15 (2.5%)	1 (0.4%)	8 (3.8%)	0 (0%)	2 (0.8%)	2 (0.8%)	28 (1.5%)
Total ISS	*M* (*SD*)	17.0 (12.1)	20.5 (17.4)	13.3 (9.94)	22.8 (18.0)	17.6 (14.7)	19.4 (16.3)	18.3 (14.8)
	*Mdn* [*Min*, *Max*]	13.0 [1.00, 75.0]	17.0 [1.00, 75.0]	9.00 [1.00, 50.0]	18.0 [1.00, 75.0]	13.0 [1.00, 75.0]	16.0 [1.00, 75.0]	13.0 [1.00, 75.0]

^a^For categorical variables and the GOSE score, absolute (N) and relative (%) frequencies are reported. Due to rounding, the values may not sum up to exactly 100%

M, mean; SD, Standard deviation; Mdn, median; Min, minimum; Max, maximum; Injury cause (Other: summarized category for relative frequencies < 5%, including non-intentional injury; suicide attempt; violence/assault, act of mass violence); ER, emergency room; ward, admission to hospital ward; ICU, intensive care unit; GOSE, Glasgow Outcome Scale – Extended; ISS, Injury Severity Score

Some significant differences were observed between the language samples regarding age, years of education, ISS, GCS at baseline, and GOSE at six months. Dutch participants were significantly older (52.9 ± 19.1) compared to all but the Italian sample. Finnish (13.3 ± 3.16) and Italian (12.4 ± 4.37) participants had slightly fewer (but statistically significant) years of education compared to the others. The Italian sample had a lower GCS (11.93 ± 4.13) compared to the Dutch, Finnish, and Norwegian samples. At six months after TBI, Finnish participants recovered slightly better (*Mdn* = 8; range 3–8) and had less severe extracranial injuries (ISS: 13.3 ± 9.94) compared to individuals from the Netherlands, the UK, Italy, and Spain (ISS only). However, the effects were small according to the predefined cut-offs (i.e., 0.35–0.44 or 0.56–0.63). The distributions of both four (i.e., uncomplicated mild, complicated mild, moderate, and severe) and two (i.e., mild/moderate and severe) TBI severity groups differed between language samples (*p* < 0.001). The effect sizes represented a small effect (V = 0.13 and V = 0.17, respectively). There was no significant difference in the RPQ total score across the samples. For details, see Additional file 1: Table S2 – Comparisons of language samples regarding sociodemographic and injury-related factors.

### Distribution of PCS across language samples and TBI severity groups

The distribution of PCS was similar across all language samples. *Fatigue* was the most frequently reported symptom at six months after TBI with 37% (Spanish sample) to 56% (English sample), followed by *Forgetfulness* with 36% (Finnish sample) to 46% (English sample), and *Poor concertation* with 31% (Spanish sample) to 40% (Italian sample). Individuals from the English sample tended to report more intense PCS (8 out of 16 symptoms were rated as at least a mild problem) compared to participants from other language samples (see Table 3, left part—Language samples).

**Table 3:** Proportion of PCS rated as at least mild

Symptom	Language samples						TBI severity	
	Dutch (N = 597) (%)	English (N = 223) (%)	Finnish (N = 213) (%)	Italian (N = 268) (%)	Norwegian (N = 263) (%)	Spanish (N = 254) (%)	Mild/moderate (N = 1,521) (%)	Severe (N = 269) (%)
Headaches	27	31	**33**	27	31	**33**	30	29
Dizziness	27	28	**34**	23	28	30	28	28
Nausea	7	8	8	7	7	6	8	4
Noise sensitivity	26	22	17	19	27	24	22	27
Sleep disturbance	29	**34**	**36**	26	28	27	30	26
Fatigue	**52**	**56**	**42**	**40**	**48**	**37**	**44**	**63**
Irritable	28	**33**	24	29	27	31	27	**36**
Depressed	27	**38**	21	28	22	28	26	**35**
Frustrated	30	**43**	23	29	28	24	28	**39**
Forgetful	**41**	**46**	**36**	**45**	**38**	**37**	**38**	**57**
Poor concentration	**37**	**39**	32	**40**	**34**	31	**34**	**44**
Longer to think	**42**	**41**	25	28	**34**	22	32	**42**
Blurred vision	19	18	18	17	18	17	17	22
Light sensitivity	14	13	16	18	16	15	15	18
Double vision	10	13	11	12	6	11	9	17
Restless	24	20	19	19	21	16	20	22

Symptoms rated at least as a mild problem (i.e., ≥ 2). Bold values indicate relative frequencies over 33% (i.e., 1/3 of each language sample)

Similar patterns were observed when examining TBI severity groups. Items *Fatigue* (44% and 63%), *Forgetfulness* (38% and 57%), and *Poor concentration* (34% and 44%) presented the most frequently reported symptoms in both the mild/moderate and severe TBI groups, respectively. More than one-third of individuals after severe TBI also rated the following symptoms as at least mild: prolonged thinking (42%), being frustrated (39%), irritable (36%), and depressed (35%) (see Table 3, right part—TBI severity). For visualization, see Additional file 2: Figure S1 – Distribution of the PCS ratings in (A) each language sample and (B) for the TBI severity groups.

### Analyses of response pattern

The analysis of response patterns per language sample revealed an unequal distribution of the response categories across all samples. Especially higher ranked categories (i.e., 3 “a moderate problem” and 4 “a severe problem”) showed a low endorsement rate in some items. One item (*Nausea*) was not rated as a severe problem in Finnish, Italian, and Norwegian samples. The frequencies of endorsements for this item in the Dutch, English, and Spanish samples were also sparse: 1 (0.4%), 2 (0.9%), 5 (0.8%). The endorsement of the category “a moderate problem” varied from 0% (English sample) to 2.2% (Dutch sample). In addition, the item *Double vision* was rated a maximum of 2 (“a mild problem”) in the Norwegian sample, resulting in no endorsement in two response categories (3 “a moderate problem” and 4 “a severe problem”). The highest endorsement rate for the category 3 “a moderate problem” or 4 “a severe problem” was observed in the Italian sample (5.2%). For more details, Additional file 1: Table S3 – Analyses of response patterns by language sample.

### Confirmatory factor analyses (CFA)

The CFA revealed that a four-factor structure [23] comprising *vertigo*, *mood/somatic*, *cognitive*, and *vision* symptoms fitted the data best across the languages closely followed by the three-factor structure [21] including *somatic*, *emotional*, and *cognitive* symptoms (see Table 4). The estimation of the two-factor model comprising emotional-somatic and cognitive domains [19] did not converge in a proper way with covariance matrix of latent variables being not positive definite. Therefore, interpretation of goodness of fit indices of this model should be carried out with caution. Correlations between latent factors were high across all models and languages (i.e., standardized coefficients exceeded 0.65; see Additional file 1: Table S4 – Correlations between latent variables (raw data)). When using trichotomized responses of the items *Nausea* and *Double Vision*, the models revealed comparable fit across the languages (i.e., difference observed on the third decimal place; see Additional file 1: Table S5 – CFA results for competitive factorial structure analyses of the RPQ across the language samples (trichotomized items Nausea and Double Vision)  for the model fit indices and Table S6 – Correlations between latent variables (trichotomized items Nausea and Double Vision) for correlations between latent factors).

**Table 4:** CFA results for competitive factorial structure analyses of the RPQ across the language samples (raw data)

Factor structure	Language	χ^2^	df	χ^2^/df	*p*	CFI	TLI	RMSEA	CI_90%_	SRMR
One-factor structure [6]	Dutch	776.54	104	7.47	< 0.001	**0.988**	**0.986**	0.105	[0.098, 0.112]	**0.075**
	English	324.13	104	3.12	< 0.001	**0.987**	**0.985**	0.098	[0.086, 0.111]	0.096
	Finnish	267.05	104	2.57	< 0.001	**0.988**	**0.986**	0.087	[**0.074**, 0.100]	0.088
	Italian	422.31	104	4.06	< 0.001	**0.979**	**0.975**	0.107	[0.097, 0.118]	0.093
	Norwegian	228.23	104	2.19	< 0.001	**0.992**	**0.991**	**0.068**	[**0.056**, 0.080]	**0.078**
	Spanish	262.18	104	2.52	< 0.001	**0.986**	**0.984**	**0.078**	[**0.066**, 0.090]	0.085
Two-factor model (emotional-somatic, cognitive)^a^ [19]	Dutch	549.25	89	6.17	< 0.001	**0.992**	**0.990**	0.094	[0.086, 0.102]	**0.065**
	English	260.63	89	2.93	< 0.001	**0.989**	**0.988**	0.094	[0.081, 0.107]	0.090
	Finnish	238.98	89	2.69	< 0.001	**0.988**	**0.986**	0.090	[**0.076**, 0.104]	0.085
	Italian	324.95	89	3.65	< 0.001	**0.983**	**0.980**	0.100	[0.088, 0.112]	0.084
	Norwegian	188.48	89	2.12	< 0.001	**0.993**	**0.992**	**0.066**	[**0.053**, **0.079**]	**0.066**
	Spanish	214.95	89	2.42	< 0.001	**0.989**	**0.987**	**0.075**	[**0.062**, 0.088]	**0.077**
Two-factor model (RPQ-3, RPQ-13) [20]	Dutch	738.87	103	7.17	< 0.001	**0.989**	**0.987**	0.103	[0.096, 0.110]	**0.071**
	English	275.66	103	2.68	< 0.001	**0.990**	**0.988**	0.087	[**0.075**, 0.100]	0.087
	Finnish	241.52	103	2.34	< 0.001	**0.990**	**0.988**	0.080	[**0.067**, 0.094]	0.083
	Italian	379.96	103	3.69	< 0.001	**0.981**	**0.978**	0.101	[0.090, 0.111]	0.086
	Norwegian	217.73	103	2.11	< 0.001	**0.993**	**0.991**	**0.065**	[**0.053**, **0.078**]	**0.076**
	Spanish	242.58	103	2.36	< 0.001	**0.987**	**0.985**	**0.073**	[**0.062**, 0.085]	**0.079**
Three-factor model (somatic, emotional, cognitive) [21]	Dutch	249.47	101	2.47	< 0.001	**0.997**	**0.997**	**0.050**	**[0.042, 0.058]**	**0.051**
	English	189.19	101	**1.87**	< 0.001	**0.995**	**0.994**	**0.063**	**[0.049, 0.077]**	**0.077**
	Finnish	158.61	101	**1.57**	< 0.001	**0.996**	**0.995**	**0.052**	**[0.036, 0.068]**	**0.067**
	Italian	239.43	101	2.37	< 0.001	**0.991**	**0.989**	**0.072**	[**0.060**, 0.084]	**0.076**
	Norwegian	128.81	101	**1.28**	0.032	**0.998**	**0.998**	**0.033**	**[0.010, 0.048]**	**0.067**
	Spanish	139.54	101	**1.38**	0.007	**0.997**	**0.996**	**0.039**	**[0.021, 0.054]**	**0.064**
Three-factor model (general somatic, mood/cognition, visual somatic) [22]	Dutch	572.62	101	5.67	< 0.001	**0.992**	**0.990**	0.089	[0.082, 0.096]	**0.060**
	English	214.40	101	2.12	< 0.001	**0.993**	**0.992**	**0.072**	[**0.058**, 0.085]	**0.074**
	Finnish	178.44	101	**1.77**	< 0.001	**0.994**	**0.993**	**0.061**	**[0.046, 0.075]**	**0.071**
	Italian	265.30	101	2.63	< 0.001	**0.989**	**0.987**	**0.078**	[**0.067**, 0.090]	**0.072**
	Norwegian	207.86	101	2.06	< 0.001	**0.993**	**0.992**	**0.064**	**[0.051, 0.076]**	**0.073**
	Spanish	165.25	101	**1.64**	< 0.001	**0.994**	**0.993**	**0.050**	**[0.036, 0.064]**	**0.067**
Four-factor model (vertigo, mood/somatic, cognitive, vision) [23]	Dutch	**176.40**	98	**1.80**	< 0.001	**0.999**	**0.998**	**0.037**	**[0.028, 0.046]**	**0.041**
	English	122.90	98	**1.25**	0.045	**0.999**	**0.998**	**0.034**	**[0.005, 0.052]**	**0.066**
	Finnish	113.21	98	**1.16**	**0.140**	**0.999**	**0.999**	**0.027**	**[0.000, 0.047]**	**0.057**
	Italian	157.42	98	**1.61**	< 0.001	**0.996**	**0.995**	**0.048**	**[0.033, 0.061]**	**0.059**
	Norwegian	117.66	98	**1.20**	**0.086**	**0.999**	**0.998**	**0.028**	**[0.000, 0.045]**	**0.062**
	Spanish	101.95	98	**1.04**	**0.372**	**1.000**	**1.000**	**0.013**	**[0.000, 0.036]**	**0.058**

Values in bold indicate good model fit according to the respective cut-offs

^a^Estimation of the two-factor model comprising emotional/somatic and cognitive domains resulted in a not positive definite covariance matrix of the latent variables. Therefore, the results should be interpreted with caution

χ^2^, chi square; df, degree of freedom; χ^2^/df, ratio (cut-off: ≤ 2); *p*, *p*-value; CFI, Comparative Fit Index (cut-off: > 0.95); TLI, Tucker-Lewis Index (cut-off: > 0.95); RMSEA, root mean square error of approximation (cut-off: < 0.08) with 90% confidence interval (CI); SRMR, standardized root mean square residual (cut-off: < 0.08)

When “1” responses were treated as zero, some fit indices indicated slightly better model fit across all estimated factorial solutions and languages. However, the item measuring *Nausea*, which was dichotomized because of missing responses in the higher response categories, showed high correlations (approx. r = 1.00) with items *Dizziness*, *Feeling Frustrated*, *Poor Concentration*, *Taking Longer to Think*, and *Blurred Vision*. Furthermore, two model estimations resulted in not positive definite covariance matrices: the three-factor model (general somatic, mood/cognition, and visual somatic symptoms) [22], and the firstly favorized four-factor model (vertigo, mood/somatic, cognitive, and vision) [23]. For details, see Additional file 1: Table S7 – CFA results for competitive factorial structure analyses of the RPQ across the language samples (considering “1” responses as “0” and using dichotomized items Nausea and Double Vision) for the model fit indices and Table S8 – Correlations between latent variables (considering “1” responses as “0” and using and dichotomized items Nausea and Double Vision) for correlations between latent factors).

Overall, the three-factor model comprising *somatic*, *emotional*, and *cognitive* factors [21] performed best across all competing factorial solutions in all language samples, regardless of how the “1” responses were treated as “1” or “0”. Therefore, this factorial solution was chosen as a baseline model for the MI analyses.

### Measurement invariance (MI)

The cross-linguistic MI analyses revealed satisfactory results (see Table 5—upper part). Except for the χ^2^ *p*-values, no fit indices exceed the predefined cut-off values in the baseline model as well as in the models with increased constraints (i.e., thresholds as well as threshold and loadings model). Model comparisons were not significant. Taken together, the free-factor model did not show any violation of measurement equivalence between languages. When treating “1” responses as zero, model fit slightly increased (see Additional file 1: Table S9 – Results of MI analyses between language samples and TBI severity groups and model comparison for the three-factor model comprising somatic, emotional, and cognitive factors considering “1” responses as “0”; upper part). Therefore, this model was considered suitable for measuring PCS using the six RPQ translations.

**Table 5:** Results of MI analyses between language samples and TBI severity groups and model comparison for the three-factor model comprising *somatic*, *emotional*, and *cognitive* factors using raw data [21]

Groups	Constrains	Model fit						Model comparison					
		χ^2^	df	*p*	CFI	TLI	RMSEA	CI_90%_	Δ χ^2^	Δdf	ΔCFI	ΔRMSEA	*p*
Language samples^a^	Baseline	1633.08	606	< 0.001	**0.976**	**0.971**	**0.075**	**[0.071, 0.080]**	–	–	–	–	–
	Thresholds	1873.67	746	< 0.001	**0.973**	**0.974**	**0.071**	**[0.067, 0.075]**	164.50	140	**0.003**	**0.004**	**0.077**
	Thresholds and loadings	1914.19	811	< 0.001	**0.974**	**0.977**	**0.068**	**[0.064, 0.071]**	71.504	65	**− 0.001**	**0.003**	**0.271**
TBI severity groups (mild/moderate vs. severe)	Baseline	1028.87	202	< 0.001	**0.98**	**0.977**	**0.068**	**[0.064, 0.072]**	–	–	–	–	–
	Thresholds	1092.79	230	< 0.001	**0.979**	**0.979**	**0.065**	**[0.061, 0.069]**	40.216	28	**0.001**	**0.003**	**0.063**
	Thresholds and loadings	1052.98	243	< 0.001	**0.981**	**0.981**	**0.061**	**[0.058, 0.065]**	10.587	13	**− 0.002**	**0.004**	**0.645**

Values in bold indicate good model fit according to the respective cut-offs

^a^Dutch, English, Finnish, Italian, Norwegian, Spanish

χ^2^, chi square; df, degree of freedom; χ^2^/df, ratio (cut-off: ≤ 2); *p*, *p*-value; CFI, Comparative Fit Index (cut-off: > 0.95); TLI, Tucker-Lewis Index (cut-off: > 0.95); RMSEA, root mean square error of approximation (cut-off: < 0.08) with 90% confidence interval (CI); Δ χ^2^, change in chi square values between compared models; Δdf, change in degrees of freedom between compared models; ΔCFI, change in CFI between compared models (cut-off: < 0.01); ΔRMSEA, change in RMSEA between compared models (cut-off: ≤ 0.01)

Analyses of the TBI severity groups revealed no violation of MI assumption as reflected by non-significant difference between the models with different constraints (see Table 5—lower part). Here, again, an increase of the model fit was observed when treating “1” as “0” (see Additional file 1: Table S9 – Results of MI analyses between language samples and TBI severity groups and model comparison for the three-factor model comprising somatic, emotional, and cognitive factors considering “1” responses as “0”; lower part). These findings also support the applicability of the three-factor solution for PCS assessment using the RPQ in both examined TBI severity groups.

### Final model

**Fig. 2:**
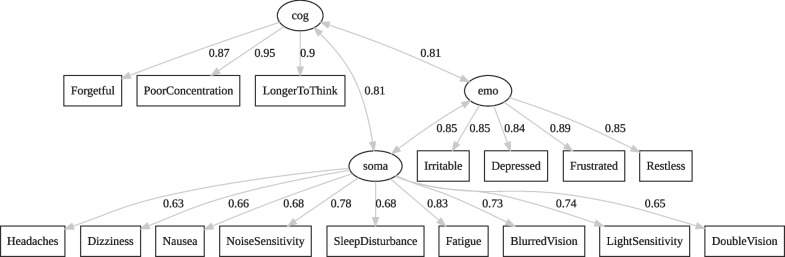
Final model including somatic (soma), emotional (emo), and cognitive (cog) factors using raw data of the total study sample. The numbers depict standardized coefficients

Estimation of the final model comprising *somatic*, *emotional*, and *cognitive* factors using raw data of the total study sample revealed satisfactory results with χ^2^(101) = 647.04, χ^2^/df = 6.41, *p* < 0.001, CFI = 0.995, TLI = 0.994, RMSEA = 0.055, CI_90%_[0.051, 0.059], SRMR = 0.051. Except for significant *p*-value and χ^2^/df-ratio > 2, which can be explained by the large sample size, all other fit indices showed excellent model fit. The correlation between latent factors was high (*somatic*–*emotional*: 0.85; *somatic*–*cognitive*: 0.81; *emotional*–*cognitive*: 0.81). For the model visualization, see Fig. 2. When treating “1” responses as “0”, the results indicated a better fit with χ^2^ (101) = 377.78, χ^2^/df = 3.74, p < 0.001, CFI = 0.997, TLI = 0.996, RMSEA = 0.039, CI_90%_[0.035, 0.043], SRMR = 0.049. Again, latent factors were highly correlated (*somatic*–*emotional*: 0.86; *somatic*–*cognitive*: 0.83; *emotional*–*cognitive*: 0.81). For the model visualization, see Additional file 3: Figure S2 – Final model somatic (soma), emotional (emo), and cognitive (cog) factors for the total study sample when treating “1” responses as “0”.

## Discussion

The present study aimed to examine the factorial validity and cross-linguistic comparability of the RPQ between six language samples. Additionally, measurement equivalence of the RPQ within TBI severity groups was investigated. The results suggest that a three-factor structure consisting of somatic, emotional, and cognitive symptom groups best captures PCS across languages. Moreover, the RPQ measures PCS equivalently across both the six language samples (i.e., Dutch, English, Finnish, Italian, Norwegian, and Spanish) and TBI severity groups (i.e., mild/moderate vs. severe). This enables national and international research on PCS and direct comparisons of outcomes across the analyzed languages within the full spectrum of TBI severity.

The RPQ has a relatively long history of attempts (2003–2018) to identify the best fitting factorial solution and thus best suitable scoring. To date, there is still no agreement as to which factorial structure would be more appropriate to assess PCS. Nevertheless, most researchers do agree on the multidimensionality of the RPQ [19–24].

Our findings show that the three-factor structure [21] including *somatic*, *emotional*, and *cognitive* scale is most appropriate for PCS assessment across six language-based samples after TBI. The favorized model is also the only one—apart from the original unidimensional factor structure—which was based on theoretical assumptions [28]. This point can also partly explain gaining problems with fitting of the models showing satisfactory results in previous studies [19, 23]. Exploratory-founded, data-driven factorial solutions may fit the data well in derivation studies but perform poorly in other datasets.

Furthermore, the scoring demonstrates clinical practicality because there are no additional constraints that may complicate the calculation of scale scores (e.g., no correlated error terms as proposed by Thomas et al. [23] to increase the model fit). In addition, two studies on factorial structure of the RPQ [19, 23], aimed in part at replicating previous scoring results, found that the three-factor model provided at least a satisfactory model fit. Potter et al. [19] found high covariance (i.e., 1.02) between the *somatic* and *emotional* latent factors, but this however was not be demonstrated in the present study (i.e., 0.85 for the total sample using raw data and 0.86 considering “1” responses as “0”).

Yielding satisfactory results in one language does not provide any evidence for cross-linguistic comparability of a questionnaire. All but one study [24], which recruited Swedish-speaking participants, investigated factorial structure of the RPQ in English-speaking samples. In the present study, we observed that fit indices of the competitive factorial solutions were comparable across the languages. Since the favored three-factor structure showed empirical evidence of MI, we would recommend using this scoring in both national and international clinical and scientific investigations using the RPQ. However, from the intercorrelations between the scales, it is evident that cognitive, somatic, and emotional symptoms are not completely independent of each other. Therefore, the use of the RPQ total score can be maintained at least as a proxy for the total PCS severity rating.

In line with previous suggestions [20], we would recommend a reduction of the response categories. In particular, the response category “1—*no more of a problem than before*” contributes hardly any added value to obtain more information. The original scoring of the RPQ excludes this category from the calculation of the total score. However, there are some pitfalls in modifying data for scoring post hoc, which is generally not recommended [44]. First, there is a difference between the number of categories presented and the number of categories used for scoring. Second, specifically in case of the RPQ, the original response scale consists of a mixture of information from the present (i.e., current symptom burden) and the past (i.e., before TBI). Although these types of scales have advantages, such as avoiding the administration of two forms of questionnaires to assess pre-TBI and post-TBI symptoms, as in the use of the Postconcussion Symptom Inventory [45], they may be particularly challenging for participants with cognitive impairments, which is likely to be the case after TBI. In addition, potential self-report or memory bias may influence response behavior in general [46] and in case of traumatic (brain) events in particular [47]. The use of this type of scale may result in inaccurate or even false information being collected. In the present study, the results of both CFAs and MI analyses using simplified response scale resulted in a better model fit. Hence, we can conclude that treating “1” as “0” may contribute to a more valid outcome assessment. However, further empirical evidence is needed before reducing the number of responses. We would suggest that future studies should address this issue by having the same group of patients complete the RPQ using different response scales (i.e., 0–4 and 0–3, where “0” could mean either “no problem at all or as before TBI” or “currently no problem”). This comparison would provide more evidence and facilitate the decision on the number of response categories of the RPQ, as has been done with other questionnaires [48].

Alternatively, future studies could address the issue of the RPQ scoring by investigating the differences between individuals choosing “0” and “1” responses, for example, using multidimensional Item Response Theory based models. Furthermore, identification of the individuals suffering from symptoms comparable to PCS prior to TBI would facilitate interpretation of the “1” responses. For example, those suffering from chronic health complains such as cancer, chronic pains, or other conditions, can suffer from fatigue, problems with concentration or sleep. This information could be considered when establishing reference or norm values for the interpretation of the results of the patients when applying the RPQ. For example, in the recent study [49] which provided reference values for the United Kingdom, the Netherlands, and Italy, one of the stratifications for the reference values was the presence the chronic health conditions which has proved important for the RPQ scores.

### Strengths and limitations

The present study holds several advantages over previous investigations. First, this is the first study involving data on multiple RPQ translations which allows for a broader overview of PCS self-report in six European languages. Second, in contrast to other studies, we applied methods within the CFA framework considering the ordinal nature of the questionnaire items. Third, we additionally address the applicability of the RPQ in different TBI severity groups which had not been yet carried out.

Some limitations should be mentioned as well. Most of the sample consisted of individuals after mild TBI. Thus, those affected by moderate and severe TBI were underrepresented in this study. Therefore, the results of the MI analyses for the TBI groups should be interpreted with caution and further investigation of moderate and severe TBI regarding PCS or PC-like symptoms is highly recommended. A larger sample size of the moderate and/or severe group may result in higher test power and thus lead to more robust results.

Furthermore, there are still some difficulties in assessing PCS related to particular symptoms. The authors are aware that modification of the responses of the items *Nausea* and *Double vision* presents a potential weakness, as response behavior reflects the exhaustion of response choices and/or absence of these symptoms at six months after TBI. Interestingly, these items have already undergone some rearrangements during previous analyses of the factorial structure of the RPQ. For example, Potter et al. [19] suggested to drop the item *Double vision* from the RPQ due to severe skewness and kurtosis. Eyres et al. [20] distinguished between “acute” and “post-acute” PCS whereby the item *Double vision* was a part of the “post-acute” scale. Other authors attributed the item either to a somatic scale [21], visual somatic [22]_,_ or visual domain [23]. Lannsjö and colleagues (2011) [24] found an underrepresentation of responses in the category “severe problem*”* in a large mild TBI sample and the omission of this item had been suggested again.

The item *Nausea* was the one with the lowest endorsement rate across all language samples. This finding is consistent with the distinction between early and late onset PCS proposed by Ryan and Warden [50] within a mild TBI group. Moreover, Eyres et al. [20] have allocated the *Nausea* item to the “acute” symptoms using a Rasch-based approach questioning the stability of the PCS and thus the factorial structure of the RPQ over time. Since our data refer to the six-month outcome assessments, there is no information on early-onset symptoms.

Furthermore, the focus of this study was on the factorial structure and its validity, as well as the comparability of the overall PCS construct across language samples. Therefore, item-by-item comparisons using differential item functioning (DIF) techniques were not conducted. Given the rigorous translation and linguistic validation process of the RPQ, which included several stages of harmonization of translations with feedback from psychologists and health professionals, translators, laypersons, and TBI patients, and item-by-item evaluation at the syntactic, cultural, idiomatic/pragmatic, and syntactic/grammatical levels, all possible linguistic issues that might arise during the translation process were addressed [10]. However, some specific problems of individual items may have been overlooked. To further strengthen the evidence for the comparability of RPQ translations, additional research involving item-level analyses is strongly encouraged.

Finally, we only took one specific point of time, i.e., six months after TBI, into account. Longitudinal analyses would provide more insight into the prevalence and persistence of PCS, and the applicability of the RPQ over time. Agtarap et al. [51] provided longitudinal analyses on PCS using a large U.S. sample of individuals after mild TBI. In Europe, a recent study using CENTER-TBI data at 3, 6, and 12 months post TBI [52] showed evidence of the applicability of the RPQ over time and the stability of the three-factor model by Smith-Seemiller et al. [21] that includes emotional, somatic, and cognitive domains.

## Conclusions

Although with some limitations, the six RPQ translations were found to measure the PCS construct equally across six European languages and TBI severity groups. The three-factor model consisting of somatic, emotional, and cognitive domains showed the best fit regardless of the treatment of “1” responses. Further studies on the reduction of the RPQ response categories may provide more insight into the comparability of four- and five-point response scales. In the absence of further evidence, we recommend the use of the three-factor structure for scoring, with “1” treated as “0”, in addition to the conventional total score. Finally, item-by-item comparisons between different translations of the RPQ are recommended to strengthen its aggregated applicability across languages.

### Supplementary Information

**Additional file 1.** Supplementary tables.

**Additional file 2.** Supplementary figures S1.

**Additional file 3.** Supplementary figures S2.

## Data Availability

All relevant data are available upon request from CENTER-TBI, and the authors are not legally allowed to share it publicly. The authors confirm that they received no special access privileges to the data. CENTER-TBI is committed to data sharing and in particular to responsible further use of the data. Hereto, we have a data sharing statement in place: https://www.center-tbi.eu/data/sharing (last access on 18 April 2023). The CENTER-TBI Management Committee, in collaboration with the General Assembly, established the Data Sharing policy, and Publication and Authorship Guidelines to assure correct and appropriate use of the data as the dataset is hugely complex and requires help of experts from the Data Curation Team or Bio- Statistical Team for correct use. This means that we encourage researchers to contact the CENTER-TBI team for any research plans and the Data Curation Team for any help in appropriate use of the data, including sharing of scripts. Requests for data access can be submitted online: https://www.center-tbi.eu/data (last access on 18 April 2023). The complete Manual for data access is also available online: https://www.center-tbi.eu/files/SOP-Manual-DAPR-2402020.pdf (assessed on 18 April 2023).

## Abbreviations

AIS: Abbreviated Injury Scale
CENTER-TBI: Collaborative European NeuroTrauma Effectiveness Research
CFA: Confirmatory factor analysis
CFI: Comparative fit index
CI: Confidence interval
CT: Computed tomography
DSM-IV/5: Diagnostic and the statistical manual of mental disorders
ER: Emergency room
GCS: Glasgow Coma Scale
GOSE: Glasgow outcome scale – extended
ICD-10/11: International classification of diseases
ICU: Intensive care unit
ISS: Injury severity score
MI: Measurement invariance
PROM: Patient-reported outcome measure
RMSEA: Root mean square error of approximation
RPQ: Rivermead Post-Concussion Symptoms Questionnaire
SRMR: Standardized root mean square residual
TBI: Traumatic brain injury
TLI: Tucker–Lewis index
WLSMV: Robust weighted least squares estimator
