# Supplementary material for: Factorial validity and comparability of the six translations of the Rivermead Post-Concussion Symptoms Questionnaire translations: results from the CENTER-TBI study
Source: J Patient Rep Outcomes. 2023 Sep 8;7:90. doi: 10.1186/s41687-023-00632-5 (PMC10491569; doi:10.1186/s41687-023-00632-5)
Supplement: Supplementary file 1 — Additional file 1. Supplementary tables. [file 41687_2023_632_MOESM1_ESM.pdf]

Table S1 Additional characteristics of the language samples.

Variable	Group/values	**Dutch** (N = 597)	**English** (N = 223)	**Finnish** (N = 213)	**Italian** (N = 268)	**Norwegian** (N = 263)	**Spanish** (N = 254)	**Total** (N = 1818)
**Education**[a]	Primary School/none/unknown	40 (6.7%)	2 (0.9%)	23 (10.8%)	64 (23.9%)	27 (10.3%)	83 (32.7%)	239 (13.1%)
	Secondary school/high school	125 (20.9%)	61 (27.4%)	69 (32.4%)	101 (37.7%)	64 (24.3%)	105 (41.3%)	525 (28.9%)
	Post-high school	363 (60.8%)	140 (62.8%)	73 (34.3%)	64 (23.9%)	161 (61.2%)	63 (24.8%)	864 (47.5%)
	Missing	69 (11.6%)	20 (9.0%)	48 (22.5%)	39 (14.6%)	11 (4.2%)	3 (1.2%)	190 (10.5%)
**Employment**[a]	Full-time employed	192 (32.2%)	119 (53.4%)	89 (41.8%)	103 (38.4%)	130 (49.4%)	130 (51.2%)	763 (42.0%)
	Part-time employed	88 (14.7%)	23 (10.3%)	7 (3.3%)	40 (14.9%)	33 (12.5%)	26 (10.2%)	217 (11.9%)
	In training	51 (8.5%)	11 (4.9%)	30 (14.1%)	27 (10.1%)	35 (13.3%)	16 (6.3%)	170 (9.4%)
	Unemployed	42 (7.0%)	18 (8.1%)	14 (6.6%)	16 (6.0%)	18 (6.8%)	23 (9.1%)	131 (7.2%)
	Retired	177 (29.6%)	38 (17.0%)	51 (23.9%)	58 (21.6%)	42 (16.0%)	54 (21.3%)	420 (23.1%)
	Missing	47 (7.9%)	14 (6.3%)	22 (10.3%)	24 (9.0%)	5 (1.9%)	5 (2.0%)	117 (6.4%)
**Marital status**[a]	Single	270 (45.2%)	109 (48.9%)	109 (51.2%)	134 (50.0%)	133 (50.6%)	133 (52.4%)	888 (48.8%)
	Partnered	325 (54.4%)	114 (51.1%)	104 (48.8%)	134 (50.0%)	130 (49.4%)	121 (47.6%)	928 (51.0%)
	Missing	2 (0.3%)	0 (0%)	0 (0%)	0 (0%)	0 (0%)	0 (0%)	2 (0.1%)
**Living situation**[a]	Alone	143 (24.0%)	47 (21.1%)	62 (29.1%)	41 (15.3%)	55 (20.9%)	37 (14.6%)	385 (21.2%)
	Not alone	454 (76.0%)	176 (78.9%)	151 (70.9%)	227 (84.7%)	208 (79.1%)	217 (85.4%)	1433 (78.8%)
**Premorbid psychiatric problems**[a]	No	532 (89.1%)	167 (74.9%)	181 (85.0%)	243 (90.7%)	224 (85.2%)	223 (87.8%)	1570 (86.4%)
	Yes	62 (10.4%)	51 (22.9%)	32 (15.0%)	25 (9.3%)	34 (12.9%)	30 (11.8%)	234 (12.9%)
	Missing	3 (0.5%)	5 (2.2%)	0 (0%)	0 (0%)	5 (1.9%)	1 (0.4%)	14 (0.8%)
**Brain Injury AIS**	*M* (*SD*)	2.82 (1.23)	2.94 (1.63)	2.84 (1.13)	3.11 (1.56)	2.97 (1.12)	2.87 (1.29)	2.91 (1.32)
	Mdn [*Min*, *Max*]	3.00 [1.00, 5.00]	3.00 [1.00, 5.00]	3.00 [1.00, 5.00]	3.00 [1.00, 5.00]	3.00 [1.00, 5.00]	3.00 [1.00, 6.00]	3.00 [1.00, 6.00]

[a] For categorical variables, absolute (N) and relative (%) frequencies are reported. Due to rounding, the values may not sum up to exactly 100%. *M* = mean, *SD* = Standard deviation, *Mdn* = median, *Min* = minimum, *Max* = maximum, Employment status = employed (full-time employed, part-time employed, on sick leave, special/sheltered employment), unemployd (looking for work, unemployed, unable to work), other (retired, student/schoolgoing, homemaker); AIS = Abbreviated Injury Scale.

Table S2 Comparisons of language samples regarding sociodemographic and injury-related factors.

	Dutch	English	Finnish	Italian	Norwegian	Spanish
**Age**						
**$\chi2(5) = 37.022$, $p < 0.001$**						
	Dutch	English	Finnish	Italian	Norwegian	Spanish
Dutch	-	0.58	0.58	0.54	0.60	0.59
English	**0.005**	-	0.51	0.47	0.54	0.53
Finnish	**0.009**	> 0.99	-	0.46	0.53	0.51
Italian	0.963	> 0.99	> 0.99	-	0.56	0.54
Norwegian	**< 0.001**	> 0.99	> 0.99	0.193	-	0.48
Spanish	**< 0.001**	> 0.99	> 0.99	> 0.99	> 0.99	-
**Years of education**						
**$\chi2(5) = 51.487$, $p < 0.001$**						
	Dutch	English	Finnish	Italian	Norwegian	Spanish
Dutch	-	0.50	0.60	0.44	0.48	0.42
English	> 0.99	-	0.61	0.44	0.48	0.41
Finnish	**0.003**	**0.001**	-	0.35	0.35	0.34
Italian	**< 0.001**	**< 0.001**	> 0.99	-	0.54	0.49
Norwegian	> 0.99	> 0.99	.067	**< 0.001**	-	0.43
Spanish	> 0.99	> 0.99	**.019**	**< 0.001**	> 0.99	-
**GCS at baseline**						
**$\chi2(5) = 37.219$, $p < 0.001$**						
	Dutch	English	Finnish	Italian	Norwegian	Spanish
Dutch	-	0.57	0.49	0.61	0.52	0.53
English	**0.042**	-	0.42	0.54	0.45	0.46
Finnish	> 0.99	0.217	-	0.62	0.52	0.53
Italian	**< 0.001**	> 0.99	**< 0.001**	-	0.40	0.43
Norwegian	> 0.99	0.356	> 0.99	**< 0.001**	-	0.51
Spanish	> 0.99	> 0.99	> 0.99	**0.044**	> 0.99	-
**GOSE at six months**						
**$\chi2(5) = 20.095$, $p < 0.001$**						
	Dutch	English	Finnish	Italian	Norwegian	Spanish
Dutch	-	0.55	0.43	0.53	0.50	0.50
English	0.428	-	0.40	0.49	0.46	0.46
Finnish	**0.024**	**0.001**	-	0.58	0.57	0.57
Italian	> 0.99	> 0.99	**0.011**	-	0.48	0.48
Norwegian	> 0.99	0.978	0.074	> 0.99	-	0.50
Spanish	> 0.99	> 0.99	0.100	> 0.99	> 0.99	-
**Total ISS**						
**$\chi2(5) = 33.977$, $p < 0.001$**						
	Dutch	English	Finnish	Italian	Norwegian	Spanish
Dutch	-	0.48	0.60	0.43	0.52	0.49
English	> 0.99	-	0.59	0.47	0.53	0.51
Finnish	**< 0.001**	**< 0.001**	-	0.36	0.42	0.41
Italian	**0.026**	> 0.99	**< 0.001**	-	0.57	0.55
Norwegian	> 0.99	> 0.99	0.061	0.083	-	0.48
Spanish	> 0.99	> 0.99	**0.006**	0.727	> 0.99	-
**RPQ total score**						
**$\chi2(5) = 6.3612$, $p = 0.273$**						
	Dutch	English	Finnish	Italian	Norwegian	Spanish
Dutch	-	0.47	0.53	0.51	0.52	0.52
English	> 0.99	-	0.55	0.54	0.55	0.55
Finnish	> 0.99	0.810	-	0.49	0.50	0.50
Italian	> 0.99	> 0.99	> 0.99	-	0.51	0.51

Norwegian	> 0.99	0.760	> 0.99	> 0.99	-	0.50
Spanish	> 0.99	0.720	> 0.99	> 0.99	> 0.99	-

Chi-square statistics are obtained from Kruskal-Wallis tests; lower table sections (grey cells) represent Bonferroni adjusted p-values for the pairwise comparisons for pairwise available observations between language samples; **bold** adjusted p-values are significant at 5%; upper table section provides Vargha and Delaney's A effect sizes, implying following cut-offs: group equality (0.50), small (0.35–0.44 or 0.56–0.63), medium (0.30–0.34 or 0.64–0.70) and large effect (beyond 0.29 or 0.71). GCS = Glasgow Coma Scale; GOSE = Glasgow Outcome Scale – Extended; ISS = Injury Severity Score; RPQ = Rivermead Post-Concussion Symptoms Questionnaire.

Table S3 Analyses of response patterns by language sample.

Item	Response	Dutch (N = 597)	English (N = 223)	Finnish (N = 213)	Italian (N = 268)	Norwegian (N = 263)	Spanish (N = 254)
**Headaches**	0	327 (54.8%)	112 (50.2%)	93 (43.7%)	138 (51.5%)	149 (56.7%)	137 (53.9%)
	1	108 (18.1%)	41 (18.4%)	49 (23.0%)	58 (21.6%)	32 (12.2%)	33 (13.0%)
	2	75 (12.6%)	36 (16.1%)	39 (18.3%)	38 (14.2%)	45 (17.1%)	43 (16.9%)
	3	55 (9.2%)	18 (8.1%)	24 (11.3%)	28 (10.4%)	34 (12.9%)	33 (13.0%)
	4	31 (5.2%)	15 (6.7%)	8 (3.8%)	6 (2.2%)	3 (1.1%)	8 (3.1%)
	Missing	1 (0.2%)	1 (0.4%)	0 (0%)	0 (0%)	0 (0%)	0 (0%)
**Dizziness**	0	334 (55.9%)	136 (61.0%)	109 (51.2%)	166 (61.9%)	160 (60.8%)	154 (60.6%)
	1	102 (17.1%)	25 (11.2%)	31 (14.6%)	41 (15.3%)	28 (10.6%)	24 (9.4%)
	2	90 (15.1%)	34 (15.2%)	45 (21.1%)	42 (15.7%)	53 (20.2%)	41 (16.1%)
	3	47 (7.9%)	21 (9.4%)	21 (9.9%)	13 (4.9%)	15 (5.7%)	24 (9.4%)
	4	24 (4.0%)	7 (3.1%)	7 (3.3%)	6 (2.2%)	6 (2.3%)	11 (4.3%)
	Missing	0 (0%)	0 (0%)	0 (0%)	0 (0%)	1 (0.4%)	0 (0%)
**Nausea**	0	507 (84.9%)	193 (86.5%)	166 (77.9%)	214 (79.9%)	230 (87.5%)	226 (89.0%)
	1	47 (7.9%)	13 (5.8%)	30 (14.1%)	36 (13.4%)	13 (4.9%)	12 (4.7%)
	2	25 (4.2%)	15 (6.7%)	16 (7.5%)	13 (4.9%)	14 (5.3%)	6 (2.4%)
	3	13 (2.2%)	0 (0%)	1 (0.5%)	5 (1.9%)	5 (1.9%)	9 (3.5%)
	4	5 (0.8%)	2 (0.9%)	**0 (0%)**	**0 (0%)**	**0 (0%)**	1 (0.4%)
	Missing	0 (0%)	0 (0%)	0 (0%)	0 (0%)	1 (0.4%)	0 (0%)
**Noise Sensitivity**	0	338 (56.6%)	149 (66.8%)	145 (68.1%)	154 (57.5%)	175 (66.5%)	164 (64.6%)
	1	104 (17.4%)	24 (10.8%)	31 (14.6%)	63 (23.5%)	17 (6.5%)	27 (10.6%)
	2	82 (13.7%)	30 (13.5%)	18 (8.5%)	26 (9.7%)	47 (17.9%)	31 (12.2%)
	3	39 (6.5%)	11 (4.9%)	14 (6.6%)	18 (6.7%)	17 (6.5%)	21 (8.3%)
	4	34 (5.7%)	9 (4.0%)	4 (1.9%)	7 (2.6%)	7 (2.7%)	10 (3.9%)
	Missing	0 (0%)	0 (0%)	1 (0.5%)	0 (0%)	0 (0%)	1 (0.4%)
**Sleep Disturbance**	0	306 (51.3%)	106 (47.5%)	91 (42.7%)	123 (45.9%)	142 (54.0%)	134 (52.8%)
	1	115 (19.3%)	41 (18.4%)	45 (21.1%)	75 (28.0%)	48 (18.3%)	50 (19.7%)
	2	76 (12.7%)	33 (14.8%)	38 (17.8%)	32 (11.9%)	39 (14.8%)	31 (12.2%)
	3	63 (10.6%)	20 (9.0%)	24 (11.3%)	23 (8.6%)	21 (8.0%)	26 (10.2%)
	4	35 (5.9%)	23 (10.3%)	15 (7.0%)	15 (5.6%)	13 (4.9%)	12 (4.7%)
	Missing	2 (0.3%)	0 (0%)	0 (0%)	0 (0%)	0 (0%)	1 (0.4%)
**Fatigue**	0	166 (27.8%)	66 (29.6%)	78 (36.6%)	93 (34.7%)	103 (39.2%)	131 (51.6%)
	1	116 (19.4%)	31 (13.9%)	46 (21.6%)	66 (24.6%)	33 (12.5%)	28 (11.0%)
	2	128 (21.4%)	54 (24.2%)	36 (16.9%)	59 (22.0%)	68 (25.9%)	40 (15.7%)
	3	123 (20.6%)	48 (21.5%)	36 (16.9%)	31 (11.6%)	42 (16.0%)	35 (13.8%)
	4	61 (10.2%)	23 (10.3%)	17 (8.0%)	18 (6.7%)	17 (6.5%)	19 (7.5%)
	Missing	3 (0.5%)	1 (0.4%)	0 (0%)	1 (0.4%)	0 (0%)	1 (0.4%)
**Irritable**	0	291 (48.7%)	107 (48.0%)	113 (53.1%)	113 (42.2%)	142 (54.0%)	139 (54.7%)
	1	137 (22.9%)	42 (18.8%)	47 (22.1%)	76 (28.4%)	49 (18.6%)	36 (14.2%)
	2	93 (15.6%)	34 (15.2%)	35 (16.4%)	28 (10.4%)	50 (19.0%)	39 (15.4%)
	3	48 (8.0%)	29 (13.0%)	10 (4.7%)	39 (14.6%)	16 (6.1%)	33 (13.0%)
	4	28 (4.7%)	11 (4.9%)	7 (3.3%)	12 (4.5%)	6 (2.3%)	7 (2.8%)
	Missing	0 (0%)	0 (0%)	1 (0.5%)	0 (0%)	0 (0%)	0 (0%)
**Depressed**	0	303 (50.8%)	106 (47.5%)	133 (62.4%)	133 (49.6%)	172 (65.4%)	146 (57.5%)
	1	134 (22.4%)	33 (14.8%)	35 (16.4%)	59 (22.0%)	32 (12.2%)	37 (14.6%)
	2	86 (14.4%)	52 (23.3%)	26 (12.2%)	34 (12.7%)	38 (14.4%)	25 (9.8%)
	3	48 (8.0%)	28 (12.6%)	15 (7.0%)	31 (11.6%)	17 (6.5%)	34 (13.4%)
	4	25 (4.2%)	4 (1.8%)	4 (1.9%)	11 (4.1%)	3 (1.1%)	12 (4.7%)
	Missing	1 (0.2%)	0 (0%)	0 (0%)	0 (0%)	1 (0.4%)	0 (0%)
**Frustrated**	0	279 (46.7%)	89 (39.9%)	118 (55.4%)	123 (45.9%)	133 (50.6%)	154 (60.6%)
	1	135 (22.6%)	39 (17.5%)	44 (20.7%)	68 (25.4%)	57 (21.7%)	38 (15.0%)
	2	95 (15.9%)	50 (22.4%)	30 (14.1%)	47 (17.5%)	41 (15.6%)	25 (9.8%)

Item	Response	Dutch (N = 597)	English (N = 223)	Finnish (N = 213)	Italian (N = 268)	Norwegian (N = 263)	Spanish (N = 254)
	3	52 (8.7%)	33 (14.8%)	17 (8.0%)	19 (7.1%)	25 (9.5%)	33 (13.0%)
	4	33 (5.5%)	12 (5.4%)	3 (1.4%)	11 (4.1%)	7 (2.7%)	4 (1.6%)
	Missing	3 (0.5%)	0 (0%)	1 (0.5%)	0 (0%)	0 (0%)	0 (0%)
**Forgetful**	0	214 (35.8%)	77 (34.5%)	86 (40.4%)	82 (30.6%)	110 (41.8%)	110 (43.3%)
	1	136 (22.8%)	43 (19.3%)	50 (23.5%)	65 (24.3%)	53 (20.2%)	51 (20.1%)
	2	122 (20.4%)	48 (21.5%)	52 (24.4%)	68 (25.4%)	60 (22.8%)	49 (19.3%)
	3	84 (14.1%)	37 (16.6%)	16 (7.5%)	35 (13.1%)	27 (10.3%)	32 (12.6%)
	4	41 (6.9%)	18 (8.1%)	9 (4.2%)	18 (6.7%)	13 (4.9%)	12 (4.7%)
	Missing	0 (0%)	0 (0%)	0 (0%)	0 (0%)	0 (0%)	0 (0%)
**Poor Concentration**	0	222 (37.2%)	96 (43.0%)	96 (45.1%)	101 (37.7%)	135 (51.3%)	133 (52.4%)
	0	222 (37.2%)	96 (43.0%)	96 (45.1%)	101 (37.7%)	135 (51.3%)	133 (52.4%)
	1	151 (25.3%)	39 (17.5%)	48 (22.5%)	59 (22.0%)	39 (14.8%)	42 (16.5%)
	2	116 (19.4%)	38 (17.0%)	34 (16.0%)	62 (23.1%)	54 (20.5%)	29 (11.4%)
	3	73 (12.2%)	33 (14.8%)	27 (12.7%)	34 (12.7%)	26 (9.9%)	38 (15.0%)
	4	34 (5.7%)	16 (7.2%)	8 (3.8%)	12 (4.5%)	9 (3.4%)	11 (4.3%)
	Missing	1 (0.2%)	1 (0.4%)	0 (0%)	0 (0%)	0 (0%)	1 (0.4%)
**Longer To Think**	0	231 (38.7%)	94 (42.2%)	125 (58.7%)	127 (47.4%)	135 (51.3%)	170 (66.9%)
	1	116 (19.4%)	36 (16.1%)	34 (16.0%)	65 (24.3%)	38 (14.4%)	26 (10.2%)
	2	132 (22.1%)	50 (22.4%)	33 (15.5%)	43 (16.0%)	55 (20.9%)	21 (8.3%)
	3	87 (14.6%)	30 (13.5%)	16 (7.5%)	24 (9.0%)	29 (11.0%)	26 (10.2%)
	4	31 (5.2%)	12 (5.4%)	4 (1.9%)	9 (3.4%)	6 (2.3%)	10 (3.9%)
	Missing	0 (0%)	1 (0.4%)	1 (0.5%)	0 (0%)	0 (0%)	1 (0.4%)
**Blurred Vision**	0	373 (62.5%)	162 (72.6%)	141 (66.2%)	175 (65.3%)	177 (67.3%)	189 (74.4%)
	1	112 (18.8%)	20 (9.0%)	32 (15.0%)	47 (17.5%)	39 (14.8%)	22 (8.7%)
	2	59 (9.9%)	23 (10.3%)	24 (11.3%)	18 (6.7%)	28 (10.6%)	16 (6.3%)
	3	37 (6.2%)	13 (5.8%)	7 (3.3%)	19 (7.1%)	16 (6.1%)	16 (6.3%)
	4	15 (2.5%)	5 (2.2%)	8 (3.8%)	9 (3.4%)	3 (1.1%)	11 (4.3%)
	Missing	1 (0.2%)	0 (0%)	1 (0.5%)	0 (0%)	0 (0%)	0 (0%)
**Light Sensitivity**	0	420 (70.4%)	166 (74.4%)	148 (69.5%)	168 (62.7%)	203 (77.2%)	184 (72.4%)
	1	91 (15.2%)	27 (12.1%)	30 (14.1%)	51 (19.0%)	17 (6.5%)	31 (12.2%)
	2	45 (7.5%)	18 (8.1%)	16 (7.5%)	23 (8.6%)	33 (12.5%)	8 (3.1%)
	3	26 (4.4%)	7 (3.1%)	13 (6.1%)	17 (6.3%)	7 (2.7%)	24 (9.4%)
	4	14 (2.3%)	4 (1.8%)	6 (2.8%)	9 (3.4%)	3 (1.1%)	7 (2.8%)
	Missing	1 (0.2%)	1 (0.4%)	0 (0%)	0 (0%)	0 (0%)	0 (0%)
**Double Vision**	0	469 (78.6%)	179 (80.3%)	179 (84.0%)	197 (73.5%)	235 (89.4%)	217 (85.4%)
	1	71 (11.9%)	15 (6.7%)	11 (5.2%)	38 (14.2%)	12 (4.6%)	9 (3.5%)
	2	27 (4.5%)	16 (7.2%)	12 (5.6%)	14 (5.2%)	16 (6.1%)	11 (4.3%)
	3	17 (2.8%)	8 (3.6%)	5 (2.3%)	14 (5.2%)	0 (0%)	9 (3.5%)
	4	13 (2.2%)	5 (2.2%)	6 (2.8%)	5 (1.9%)	0 (0%)	7 (2.8%)
	Missing	0 (0%)	0 (0%)	0 (0%)	0 (0%)	0 (0%)	1 (0.4%)
**Restless**	0	323 (54.1%)	136 (61.0%)	130 (61.0%)	160 (59.7%)	170 (64.6%)	186 (73.2%)
	1	131 (21.9%)	42 (18.8%)	43 (20.2%)	57 (21.3%)	38 (14.4%)	28 (11.0%)
	2	78 (13.1%)	26 (11.7%)	25 (11.7%)	29 (10.8%)	36 (13.7%)	20 (7.9%)
	3	43 (7.2%)	15 (6.7%)	10 (4.7%)	15 (5.6%)	12 (4.6%)	13 (5.1%)
	4	21 (3.5%)	4 (1.8%)	5 (2.3%)	7 (2.6%)	7 (2.7%)	7 (2.8%)
	Missing	1 (0.2%)	0 (0%)	0 (0%)	0 (0%)	0 (0%)	0 (0%)

0 = not experienced at all, 1 = no more of a problem than before, 2 = a mild problem, 3 = a moderate problem, 4 = a severe problem. Numbers in cells represent count (*N*) and percentage (%) per response category and language sample. **Bold** values indicate categories with zero responses.

Table S4 Correlations between latent variables (raw data).

Factor structure	Language	Correlated latent variables	Estimate	S.E.
**Two-factor model (emotional/somatic, cognitive) [1][a]**	Dutch	emotional/somatic – cognitive	0.918	0.011
	English	emotional/somatic – cognitive	0.936	0.016
	Finnish	emotional/somatic – cognitive	1.002	0.016
	Italian	emotional/somatic – cognitive	0.914	0.018
	Norwegian	emotional/somatic – cognitive	0.939	0.015
	Spanish	emotional/somatic – cognitive	0.933	0.023
**Two-factor model (RPQ-3, RPQ-13) [2]**	Dutch	RPQ-3 – RPQ-13	0.833	0.027
	English	RPQ-3 – RPQ-13	0.754	0.049
	Finnish	RPQ-3 – RPQ-13	0.768	0.043
	Italian	RPQ-3 – RPQ-13	0.709	0.047
	Norwegian	RPQ-3 – RPQ-13	0.877	0.030
	Spanish	RPQ-3 – RPQ-13	0.790	0.044
**Three-factor model (somatic, emotional, cognitive) [3]**	Dutch	somatic – emotional	0.862	0.016
		somatic – cognitive	0.826	0.019
		emotional – cognitive	0.787	0.020
	English	somatic – emotional	0.829	0.031
		somatic – cognitive	0.822	0.028
		emotional – cognitive	0.804	0.032
	Finnish	somatic – emotional	0.817	0.039
		somatic – cognitive	0.791	0.037
		emotional – cognitive	0.913	0.022
	Italian	somatic – emotional	0.876	0.022
		somatic – cognitive	0.761	0.033
		emotional – cognitive	0.784	0.030
	Norwegian	somatic – emotional	0.873	0.025
		somatic – cognitive	0.869	0.024
		emotional – cognitive	0.809	0.032
	Spanish	somatic – emotional	0.827	0.033
		somatic – cognitive	0.774	0.037
		emotional – cognitive	0.788	0.034
**Three-factor model (general somatic, mood/cognition, visual somatic) [4]**	Dutch	general somatic – mood/cognition	0.938	0.011
		general somatic – visual somatic	0.819	0.027
		mood/cognition – visual somatic	0.720	0.027
	English	general somatic – mood/cognition	0.832	0.029
		general somatic – visual somatic	0.757	0.054
		mood/cognition – visual somatic	0.678	0.053
	Finnish	general somatic – mood/cognition	0.957	0.020
		general somatic – visual somatic	0.861	0.036
		mood/cognition – visual somatic	0.705	0.052
	Italian	general somatic – mood/cognition	0.833	0.025
		general somatic – visual somatic	0.726	0.047
		mood/cognition – visual somatic	0.696	0.043
	Norwegian	general somatic – mood/cognition	0.947	0.018
		general somatic – visual somatic	0.876	0.050
		mood/cognition – visual somatic	0.813	0.046
	Spanish	general somatic – mood/cognition	0.907	0.020
		general somatic – visual somatic	0.834	0.043
		mood/cognition – visual somatic	0.691	0.048
**Four-factor model (vertigo, mood/somatic, cognitive, vision) [5]**	Dutch	vertigo – mood	0.869	0.022
		vertigo – cognition	0.804	0.026
		vertigo – vision	0.815	0.03
		mood – cognition	0.811	0.019

Factor structure	Language	Correlated latent variables	Estimate	S.E.
		mood - vision	0.715	0.03
		cognition – vision	0.715	0.030
	English	vertigo – mood	0.760	0.047
		vertigo – cognition	0.780	0.04
		vertigo – vision	0.718	0.064
		mood – cognition	0.811	0.031
		mood - vision	0.670	0.056
		cognition – vision	0.686	0.050
	Finnish	vertigo – mood	0.809	0.044
		vertigo – cognition	0.755	0.049
		vertigo – vision	0.859	0.042
		mood – cognition	0.913	0.020
		mood - vision	0.711	0.052
		cognition – vision	0.656	0.059
	Italian	vertigo – mood	0.887	0.032
		vertigo – cognition	0.641	0.05
		vertigo – vision	0.659	0.06
		mood – cognition	0.811	0.027
		mood - vision	0.726	0.043
		cognition – vision	0.659	0.052
	Norwegian	vertigo – mood	0.870	0.026
		vertigo – cognition	0.793	0.037
		vertigo – vision	0.877	0.053
		mood – cognition	0.849	0.027
		mood - vision	0.758	0.051
		cognition – vision	0.816	0.050
	Spanish	vertigo – mood	0.852	0.038
		vertigo – cognition	0.771	0.044
		vertigo – vision	0.792	0.046
		mood – cognition	0.816	0.031
		mood - vision	0.753	0.045
		cognition – vision	0.622	0.058

[a] Standardized estimates of the two-factor model can exceed 1 because the covariance matrix of latent variables was not positive definite. The results should be interpreted with caution.
Estimate = standardized coefficient reflecting correlation between laten variables, S.E. = standard error.

Table S5 CFA results for competitive factorial structure analyses of the RPQ across the language samples (trichotomized items Nausea and Double Vision).

Factor structure	Language	$\chi2$	df	$\chi2$/df	*p*	CFI	TLI	RMSEA	$CI_{90\%}$	SRMR
**One-factor structure [6]**	Dutch	776.54	104	7.47	< 0.001	**0.988**	**0.986**	0.105	[0.098, 0.112]	**0.075**
	English	324.13	104	3.12	< 0.001	**0.987**	**0.985**	0.098	[0.086, 0.111]	0.096
	Finnish	267.05	104	2.57	< 0.001	**0.988**	**0.986**	0.087	[**0.074**, 0.100]	0.088
	Italian	422.31	104	4.06	< 0.001	**0.979**	**0.975**	0.107	[0.097, 0.118]	0.093
	Norwegian	228.23	104	2.19	< 0.001	**0.992**	**0.991**	**0.068**	[**0.056**, 0.080]	**0.078**
	Spanish	262.18	104	2.52	< 0.001	**0.986**	**0.984**	**0.078**	[**0.066**, 0.090]	0.085
**Two-factor model (emotional-somatic, cognitive)[a] [1]**	Dutch	549.25	89	6.17	< 0.001	**0.992**	**0.990**	0.094	[0.086, 0.102]	**0.065**
	English	260.63	89	2.93	< 0.001	**0.989**	**0.988**	0.094	[0.081, 0.107]	0.090
	Finnish	238.98	89	2.69	< 0.001	**0.988**	**0.986**	0.090	[**0.076**, 0.104]	0.085
	Italian	324.95	89	3.65	< 0.001	**0.983**	**0.980**	0.100	[0.088, 0.112]	0.084
	Norwegian	188.48	89	2.12	< 0.001	**0.993**	**0.992**	**0.066**	[**0.053**, **0.079**]	**0.066**
	Spanish	214.95	89	2.42	< 0.001	**0.989**	**0.987**	**0.075**	[**0.062**, 0.088]	**0.077**
**Two-factor model (RPQ-3, RPQ-13) [2]**	Dutch	738.87	103	7.17	< 0.001	**0.989**	**0.987**	0.103	[0.096, 0.110]	**0.071**
	English	275.66	103	2.68	< 0.001	**0.990**	**0.988**	0.087	[**0.075**, 0.100]	0.087
	Finnish	241.52	103	2.34	< 0.001	**0.990**	**0.988**	0.080	[**0.067**, 0.094]	0.083
	Italian	379.96	103	3.69	< 0.001	**0.981**	**0.978**	0.101	[0.090, 0.111]	0.086
	Norwegian	217.73	103	2.11	< 0.001	**0.993**	**0.991**	**0.065**	[**0.053**, **0.078**]	**0.076**
	Spanish	242.58	103	2.36	< 0.001	**0.987**	**0.985**	**0.073**	[**0.062**, 0.085]	**0.079**
**Three-factor model (somatic, emotional, cognitive) [3]**	Dutch	249.47	101	2.47	< 0.001	**0.997**	**0.997**	**0.050**	**[0.042, 0.058]**	**0.051**
	English	189.19	101	**1.87**	< 0.001	**0.995**	**0.994**	**0.063**	**[0.049, 0.077]**	**0.077**
	Finnish	158.61	101	**1.57**	< 0.001	**0.996**	**0.995**	**0.052**	**[0.036, 0.068]**	**0.067**
	Italian	239.43	101	2.37	< 0.001	**0.991**	**0.989**	**0.072**	[**0.060**, 0.084]	**0.076**
	Norwegian	128.81	101	**1.28**	0.032	**0.998**	**0.998**	**0.033**	**[0.010, 0.048]**	**0.067**
	Spanish	139.54	101	**1.38**	0.007	**0.997**	**0.996**	**0.039**	**[0.021, 0.054]**	**0.064**
**Three-factor model (general somatic, mood/cognition, visual somatic) [4]**	Dutch	572.62	101	5.67	< 0.001	**0.992**	**0.990**	0.089	[0.082, 0.096]	**0.060**
	English	214.40	101	2.12	< 0.001	**0.993**	**0.992**	**0.072**	[**0.058**, 0.085]	**0.074**
	Finnish	178.44	101	**1.77**	< 0.001	**0.994**	**0.993**	**0.061**	**[0.046, 0.075]**	**0.071**
	Italian	265.30	101	2.63	< 0.001	**0.989**	**0.987**	**0.078**	[**0.067**, 0.090]	**0.072**
	Norwegian	207.86	101	2.06	< 0.001	**0.993**	**0.992**	**0.064**	**[0.051, 0.076]**	**0.073**
	Spanish	165.25	101	**1.64**	< 0.001	**0.994**	**0.993**	**0.050**	**[0.036, 0.064]**	**0.067**
**Four-factor model (vertigo, mood/somatic, cognitive, vision) [5]**	Dutch	**176.40**	98	**1.80**	< 0.001	**0.999**	**0.998**	**0.037**	**[0.028, 0.046]**	**0.041**
	English	122.90	98	**1.25**	0.045	**0.999**	**0.998**	**0.034**	**[0.005, 0.052]**	**0.066**
	Finnish	113.21	98	**1.16**	**0.140**	**0.999**	**0.999**	**0.027**	**[0.000, 0.047]**	**0.057**
	Italian	157.42	98	**1.61**	< 0.001	**0.996**	**0.995**	**0.048**	**[0.033, 0.061]**	**0.059**
	Norwegian	117.66	98	**1.20**	**0.086**	**0.999**	**0.998**	**0.028**	**[0.000, 0.045]**	**0.062**
	Spanish	101.95	98	**1.04**	**0.372**	**1.000**	**1.000**	**0.013**	**[0.000, 0.036]**	**0.058**

[a] Estimation of the two-factor model comprising emotional/somatic and cognitive domains resulted in a non-positive definite covariance matrix of the latent variables. Therefore, the results should be interpreted with caution. $\chi2$ = chi square, df = degree of freedom, $\chi2$/df = ratio (cut-off: ≤ 2), p = *p*-value, CFI = Comparative Fit Index (cut-off: > 0.95), TLI = Tucker-Lewis Index (cut-off: > 0.95), RMSEA = root mean square error of approximation (cut-off: < 0.08) with 90% confidence interval (CI), SRMR = standardized root mean square residual (cut-off: < 0.08). Values in **bold** indicate good model fit accoring to the respective cut-offs.

Table S6 Correlations between latent variables (trichotomized items Nausea and Double Vision).

Factor structure	Language	Correlated latent variables	Estimate	S.E.
**Two-factor model (emotional/somatic, cognitive) [1][a]**	Dutch	emotional/somatic – cognitive	0.918	0.011
	English	emotional/somatic – cognitive	0.935	0.016
	Finnish	emotional/somatic – cognitive	1.001[1]	0.016
	Italian	emotional/somatic – cognitive	0.915	0.018
	Norwegian	emotional/somatic – cognitive	0.939	0.015
	Spanish	emotional/somatic – cognitive	0.934	0.023
**Two-factor model (RPQ-3, RPQ-13) [2]**	Dutch	RPQ-3 – RPQ-13	0.833	0.028
	English	RPQ-3 – RPQ-13	0.757	0.050
	Finnish	RPQ-3 – RPQ-13	0.774	0.044
	Italian	RPQ-3 – RPQ-13	0.718	0.049
	Norwegian	RPQ-3 – RPQ-13	0.879	0.032
	Spanish	RPQ-3 – RPQ-13	0.787	0.044
**Three-factor model (somatic, emotional, cognitive) [3]**	Dutch	somatic – emotional	0.862	0.016
		somatic – cognitive	0.826	0.019
		emotional – cognitive	0.787	0.020
	English	somatic – emotional	0.829	0.031
		somatic – cognitive	0.822	0.028
		emotional – cognitive	0.804	0.032
	Finnish	somatic – emotional	0.817	0.039
		somatic – cognitive	0.791	0.037
		emotional – cognitive	0.913	0.022
	Italian	somatic – emotional	0.876	0.022
		somatic – cognitive	0.761	0.033
		emotional – cognitive	0.784	0.030
	Norwegian	somatic – emotional	0.873	0.025
		somatic – cognitive	0.869	0.024
		emotional – cognitive	0.809	0.032
	Spanish	somatic – emotional	0.827	0.033
		somatic – cognitive	0.774	0.037
		emotional – cognitive	0.788	0.034
**Three-factor model (general somatic, mood/cognition, visual somatic) [4]**	Dutch	general somatic – mood/cognition	0.939	0.011
		general somatic – visual somatic	0.820	0.027
		mood/cognition – visual somatic	0.721	0.028
	English	general somatic – mood/cognition	0.834	0.029
		general somatic – visual somatic	0.763	0.055
		mood/cognition – visual somatic	0.687	0.054
	Finnish	general somatic – mood/cognition	0.961	0.021
		general somatic – visual somatic	0.868	0.036
		mood/cognition – visual somatic	0.701	0.053
	Italian	general somatic – mood/cognition	0.835	0.025
		general somatic – visual somatic	0.724	0.047
		mood/cognition – visual somatic	0.694	0.043
	Norwegian	general somatic – mood/cognition	0.949	0.018
		general somatic – visual somatic	0.878	0.050
		mood/cognition – visual somatic	0.813	0.046
	Spanish	general somatic – mood/cognition	0.916	0.020
		general somatic – visual somatic	0.850	0.046
		mood/cognition – visual somatic	0.696	0.048
**Four-factor model (vertigo, mood/somatic, cognitive, vision) [5]**	Dutch	vertigo – mood	0.871	0.023
		vertigo – cognition	0.806	0.026
		vertigo – vision	0.820	0.03
		mood – cognition	0.811	0.019

Factor structure	Language	Correlated latent variables	Estimate	S.E.
		mood – vision	0.714	0.03
		cognition – vision	0.708	0.032
	English	vertigo – mood	0.765	0.046
		vertigo – cognition	0.782	0.041
		vertigo – vision	0.724	0.067
		mood – cognition	0.811	0.031
		mood – vision	0.681	0.055
		cognition – vision	0.695	0.06
	Finnish	vertigo – mood	0.808	0.045
		vertigo – cognition	0.755	0.049
		vertigo – vision	0.858	0.042
		mood – cognition	0.913	0.020
		mood - vision	0.709	0.053
		cognition – vision	0.651	0.060
	Italian	vertigo – mood	0.891	0.032
		vertigo – cognition	0.647	0.051
		vertigo – vision	0.659	0.06
		mood – cognition	0.811	0.027
		mood – vision	0.724	0.043
		cognition – vision	0.658	0.053
	Norwegian	vertigo – mood	0.871	0.027
		vertigo – cognition	0.796	0.037
		vertigo – vision	0.878	0.053
		mood – cognition	0.849	0.027
		mood – vision	0.758	0.051
		cognition – vision	0.816	0.050
	Spanish	vertigo – mood	0.865	0.039
		vertigo – cognition	0.782	0.042
		vertigo – vision	0.808	0.051
		mood – cognition	0.816	0.031
		mood – vision	0.760	0.045
		cognition – vision	0.629	0.058

[a] Standardized estimates of the two-factor model can exceed 1 because the covariance matrix of latent variables was not positive definite. The results should be interpreted with caution. Estimate = standardized coefficient reflecting correlation between laten variables, S.E. = standard error.

Table S7 CFA results for competitive factorial structure analyses of the RPQ across the language samples (considering “1” responses as “0” and using dichotomized items Nausea and Double Vision).

Factor structure	Language	$\chi2$	df	$\chi2$/df	*p*	CFI	TLI	RMSEA	$CI_{90\%}$	SRMR
**One-factor structure [6]**	Dutch	480.58	104	4.62	< 0.001	**0.989**	**0.987**	**0.079**	[**0.072**, 0.086]	0.082
	English	195.76	104	**1.88**	< 0.001	**0.994**	**0.993**	**0.063**	**[0.050, 0.077]**	0.124
	Finnish	172.05	104	**1.65**	< 0.001	**0.994**	**0.993**	**0.056**	**[0.041, 0.071]**	0.390
	Italian	240.59	104	2.31	< 0.001	**0.989**	**0.987**	**0.070**	[**0.059**, 0.082]	0.103
	Norwegian	166.71	104	**1.60**	< 0.001	**0.995**	**0.994**	**0.048**	**[0.034, 0.061]**	0.087
	Spanish	186.74	104	**1.80**	< 0.001	**0.992**	**0.991**	**0.056**	**[0.043, 0.069]**	0.087
**Two-factor model (emotional-somatic, cognitive) [1]**	Dutch	384.67	89	4.32	< 0.001	**0.991**	**0.99**	**0.075**	[**0.068**, 0.083]	**0.076**
	English	151.38	89	**1.70**	< 0.001	**0.996**	**0.995**	**0.057**	**[0.041, 0.072]**	0.125
	Finnish	148.62	89	**1.67**	< 0.001	**0.994**	**0.993**	**0.057**	**[0.040, 0.072]**	0.400
	Italian	197.39	89	2.22	< 0.001	**0.991**	**0.989**	**0.068**	[**0.055**, 0.080]	0.099
	Norwegian	141.62	89	**1.59**	< 0.001	**0.996**	**0.995**	**0.048**	**[0.032, 0.062]**	**0.078**
	Spanish	149.48	89	**1.68**	< 0.001	**0.994**	**0.993**	**0.052**	**[0.037, 0.066]**	**0.078**
**Two-factor model (RPQ-3, RPQ-13) [2]**	Dutch	457.88	103	4.45	< 0.001	**0.990**	**0.988**	**0.077**	[**0.070**, 0.084]	**0.077**
	English	187.44	103	**1.82**	< 0.001	**0.994**	**0.993**	**0.061**	**[0.047, 0.075]**	0.118
	Finnish	165.47	103	**1.61**	< 0.001	**0.994**	**0.993**	**0.054**	**[0.038, 0.069]**	0.384
	Italian	233.32	103	2.27	< 0.001	**0.989**	**0.988**	**0.069**	[**0.057**, 0.081]	0.098
	Norwegian	164.46	103	**1.60**	< 0.001	**0.995**	**0.994**	**0.048**	**[0.034, 0.061]**	0.086
	Spanish	179.54	103	**1.74**	< 0.001	**0.993**	**0.992**	**0.054**	**[0.041, 0.067]**	0.083
**Three-factor model (somatic, emotional, cognitive) [3]**	Dutch	152.07	101	**1.51**	0.001	**0.999**	**0.998**	**0.029**	**[0.019, 0.039]**	**0.054**
	English	150.87	101	**1.49**	0.001	**0.997**	**0.996**	**0.047**	**[0.031, 0.063]**	0.081
	Finnish	111.64	101	**1.11**	**0.220**	**0.999**	**0.999**	**0.023**	**[0.000, 0.044]**	**0.073**
	Italian	136.34	101	**1.35**	0.011	**0.997**	**0.997**	**0.036**	**[0.018, 0.051]**	**0.077**
	Norwegian	97.82	101	**0.97**	**0.571**	**1.000**	**1.000**	**0.000**	**[0.000, 0.030]**	**0.069**
	Spanish	120.16	101	**1.19**	**0.094**	**0.998**	**0.998**	**0.027**	**[0.000, 0.045]**	**0.070**
**Three-factor model (general somatic, mood/cognition, visual somatic)[a] [4]**	Dutch	390.35	101	3.86	< 0.001	**0.992**	**0.990**	**0.070**	**[0.063, 0.077]**	**0.070**
	English	150.73	101	**1.49**	0.001	**0.997**	**0.996**	**0.047**	**[0.031, 0.063]**	0.122
	Finnish	116.59	101	**1.15**	0.138	**0.999**	**0.998**	**0.027**	**[0.000, 0.047]**	0.384
	Italian	172.58	101	**1.71**	< 0.001	**0.994**	**0.993**	**0.052**	**[0.038, 0.065]**	0.089
	Norwegian	157.98	101	**1.56**	< 0.001	**0.996**	**0.995**	**0.047**	**[0.032, 0.060]**	0.086
	Spanish	127.56	101	**1.26**	0.038	**0.998**	**0.997**	**0.032**	**[0.008, 0.048]**	**0.074**
**Four-factor model (vertigo, mood/somatic, cognitive, vision)[a] [5]**	Dutch	122.38	98	**1.25**	0.048	**0.999**	**0.999**	**0.021**	[**0.002**, **0.031**]	**0.045**
	English	102.25	98	**1.04**	**0.365**	**1.000**	**1.000**	**0.014**	[**0.000**, **0.039**]	**0.070**
	Finnish	89.27	98	**0.91**	**0.724**	**1.000**	**1.001**	**0.000**	[**0.000**, **0.029**]	**0.065**
	Italian	97.46	98	**0.99**	**0.496**	**1.000**	**1.000**	**0.000**	[**0.000**, **0.032**]	**0.061**
	Norwegian	95.00	98	**0.97**	**0.567**	**1.000**	**1.000**	**0.000**	[**0.000**, **0.030**]	**0.069**
	Spanish	95.71	98	**0.98**	**0.547**	**1.000**	**1.000**	**0.000**	**[0.000, 0.032]**	**0.063**

[a] The results should be interpreted with caution because the covariance matrix of latent variables was not positive definite. $\chi2$ = chi square, df = degree of freedom, $\chi2$/df = ratio (cut-off: ≤ 2), p = *p*-value, CFI = Comparative Fit Index (cut-off: > 0.95), TLI = Tucker-Lewis Index (cut-off: > 0.95), RMSEA = root mean square error of approximation (cut-off: < 0.08) with 90% confidence interval (CI), SRMR = standardized root mean square residual (cut-off: < 0.08). Values in **bold** indicate good model fit accoring to the respective cut-offs.

Table S8 Correlations between latent variables (considering "1" responses as "0" and using and dichotomized items Nausea and Double Vision).

Factor structure	Language	Correlated latent variables	Estimate	S.E.
**Two-factor model (emotional/somatic, cognitive) [1]**	Dutch	emotional/somatic – cognitive	0.922	0.013
	English	emotional/somatic – cognitive	0.954	0.016
	Finnish	emotional/somatic – cognitive	0.980	0.017
	Italian	emotional/somatic – cognitive	0.932	0.019
	Norwegian	emotional/somatic – cognitive	0.940	0.018
	Spanish	emotional/somatic – cognitive	0.934	0.025
**Two-factor model (RPQ-3, RPQ-13) [2]**	Dutch	RPQ-3 – RPQ-13	0.835	0.039
	English	RPQ-3 – RPQ-13	0.820	0.060
	Finnish	RPQ-3 – RPQ-13	0.820	0.060
	Italian	RPQ-3 – RPQ-13	0.762	0.071
	Norwegian	RPQ-3 – RPQ-13	0.910	0.042
	Spanish	RPQ-3 – RPQ-13	0.832	0.048
**Three-factor model (somatic, emotional, cognitive) [3]**	Dutch	somatic – emotional	0.849	0.021
		somatic – cognitive	0.824	0.022
		emotional – cognitive	0.777	0.027
	English	somatic – emotional	0.857	0.035
		somatic – cognitive	0.844	0.029
		emotional – cognitive	0.822	0.037
	Finnish	somatic – emotional	0.835	0.045
		somatic – cognitive	0.810	0.044
		emotional – cognitive	0.935	0.025
	Italian	somatic – emotional	0.917	0.024
		somatic – cognitive	0.814	0.034
		emotional – cognitive	0.798	0.036
	Norwegian	somatic – emotional	0.906	0.028
		somatic – cognitive	0.854	0.030
		emotional – cognitive	0.822	0.038
	Spanish	somatic – emotional	0.844	0.035
		somatic – cognitive	0.834	0.034
		emotional – cognitive	0.801	0.041
**Three-factor model (general somatic, mood/cognition, visual somatic)[a] [4]**	Dutch	general somatic – mood/cognition	0.953	0.014
		general somatic – visual somatic	0.847	0.038
		mood/cognition – visual somatic	0.728	0.036
	English	general somatic – mood/cognition	0.902	0.031
		general somatic – visual somatic	0.786	0.064
		mood/cognition – visual somatic	0.725	0.060
	Finnish	general somatic – mood/cognition	0.982	0.023
		general somatic – visual somatic	0.864	0.045
		mood/cognition – visual somatic	0.710	0.067
	Italian	general somatic – mood/cognition	0.870	0.028
		general somatic – visual somatic	0.778	0.054
		mood/cognition – visual somatic	0.754	0.049
	Norwegian	general somatic – mood/cognition	0.957	0.021
		general somatic – visual somatic	0.991	0.057
		mood/cognition – visual somatic	0.905	0.048
	Spanish	general somatic – mood/cognition	0.939	0.021
		general somatic – visual somatic	0.894	0.047
		mood/cognition – visual somatic	0.758	0.051
**Four-factor model (vertigo, mood/somatic, cognitive,**	Dutch	vertigo – mood	0.853	0.031
		vertigo – cognition	0.816	0.029
		vertigo – vision	0.842	0.041

Factor structure	Language	Correlated latent variables	Estimate	S.E.
**vision)**[a]		mood – cognition	0.800	0.025
**[5]**		mood – vision	0.710	0.040
		cognition – vision	0.720	0.041
	English	vertigo – mood	0.817	0.056
		vertigo – cognition	0.869	0.046
		vertigo – vision	0.751	0.081
		mood – cognition	0.826	0.034
		mood – vision	0.709	0.066
		cognition – vision	0.732	0.065
	Finnish	vertigo – mood	0.857	0.056
		vertigo – cognition	0.772	0.071
		vertigo – vision	0.866	0.052
		mood – cognition	0.931	0.023
		mood – vision	0.710	0.067
		cognition – vision	0.683	0.074
	Italian	vertigo – mood	0.924	0.042
		vertigo – cognition	0.693	0.063
		vertigo – vision	0.704	0.077
		mood – cognition	0.833	0.031
		mood – vision	0.786	0.049
		cognition – vision	0.714	0.058
	Norwegian	vertigo – mood	0.909	0.026
		vertigo – cognition	0.782	0.046
		vertigo – vision	0.996	0.059
		mood – cognition	0.857	0.032
		mood – vision	0.895	0.049
		cognition – vision	0.855	0.056
	Spanish	vertigo – mood	0.879	0.043
		vertigo – cognition	0.874	0.040
		vertigo – vision	0.862	0.057
		mood – cognition	0.833	0.035
		mood – vision	0.799	0.047
		cognition – vision	0.719	0.061

[a] The results should be interpreted with caution because the covariance matrix of latent variables was not positive definite. Estimate = standardized coefficient reflecting correlation between laten variables, S.E. = standard error.

Table S9 Results of MI analyses between language samples and TBI severity groups and model comparison for the three-factor model comprising somatic, emotional, and cognitive factors considering “1” responses as “0”.

		Model fit							Model comparison				
**Groups**	Constrains	$\chi2$	df	*p*	CFI	TLI	RMSEA	$CI_{90\%}$	$\Delta\chi2$	Δdf	ΔCFI	ΔRMSEA	*p*
**Language samples[a]**	baseline	1123.10	606	< 0.001	**0.984**	**0.981**	**0.053**	**[0.049, 0.058]**	-	-	-	-	-
	tresholds	1217.45	676	< 0.001	**0.983**	**0.982**	**0.052**	**[0.047, 0.056]**	56.108	70	**0.001**	**0.001**	**0.886**
	thresholds and loadings	1261.85	741	< 0.001	**0.984**	**0.985**	**0.049**	**[0.044, 0.053]**	50.916	65	**-0.001**	**0.003**	**0.899**
**TBI severity groups groups (mild/moderate vs. severe)**	baseline	665.99	202	< 0.001	**0.986**	**0.983**	**0.051**	**[0.047, 0.055]**	-	-	-	-	-
	tresholds	682.94	216	< 0.001	**0.986**	**0.984**	**0.049**	**[0.045, 0.054]**	7.101	14	-	-	**0.931**
	thresholds and loadings	675.75	229	< 0.001	**0.986**	**0.986**	**0.047**	**[0.043, 0.051]**	9.682	13	**0.000**	**0.002**	**0.720**

[a] Dutch, English, Finnish, Italian, Norwegian, Spanish. $\chi2$ = chi square, df = degree of freedom, $\chi2$/df = ratio (cut-off: ≤ 2), p = p-value, CFI = Comparative Fit Index (cut-off: > 0.95), TLI = Tucker-Lewis Index (cut-off: > 0.95), RMSEA = root mean square error of approximation (cut-off: < 0.08) with 90% confidence interval (CI), $\Delta\chi2$ = change in chi square values between compared models, Δdf = change in degrees of freedom between comapred models, ΔCFI = change in CFI between compared models (cut-off: < 0.01), ΔRMSEA = change in RMSEA between compared models (cut-off: ≤ 0.01). Values in **bold** indicate good model fit accoring to the respective cut-offs.

References

1. Potter S, Leigh E, Wade D, Fleminger S (2006) The Rivermead Post Concussion Symptoms Questionnaire: A confirmatory factor analysis. J Neurol 253:1603–1614. https://doi.org/10.1007/s00415-006-0275-z

2. Eyres S, Carey A, Gilworth G, et al (2005) Construct validity and reliability of the Rivermead Post-Concussion Symptoms Questionnaire. Clin Rehabil 19:878–887. https://doi.org/10.1191/0269215505cr905oa

3. Smith-Seemiller L, Fow NR, Kant R, Franzen MD (2003) Presence of post-concussion syndrome symptoms in patients with chronic pain vs mild traumatic brain injury. Brain Injury 17:199–206. https://doi.org/10.1080/0269905021000030823

4. Herrmann N, Rapoport MJ, Rajaram RD, et al (2009) Factor Analysis of the Rivermead Post-Concussion Symptoms Questionnaire in Mild-to-Moderate Traumatic Brain Injury Patients. JNP 21:181–188. https://doi.org/10.1176/jnp.2009.21.2.181

5. Thomas M, Skilbeck C, Cannan P, Slatyer M (2018) The Structure of the Rivermead Post-Concussion Symptoms Questionnaire in Australian Adults with Traumatic Brain Injury. Brain Impairment 19:166–182. https://doi.org/10.1017/BrImp.2017.26

6. King NS, Crawford S, Wenden FJ, et al (1995) The Rivermead Post Concussion Symptoms Questionnaire: a measure of symptoms commonly experienced after head injury and its reliability. J Neurol 242:587–592
