# Supplementary material for: Factorial validity and comparability of the six translations of the Rivermead Post-Concussion Symptoms Questionnaire translations: results from the CENTER-TBI study
Source: J Patient Rep Outcomes. 2023 Sep 8;7:90. doi: 10.1186/s41687-023-00632-5 (PMC10491569; doi:10.1186/s41687-023-00632-5)

Fig. S1

Distribution of the PCS ratings in (A) each language sample and (B) for the TBI severity groups. Red symbols indicate symptoms rated above 1 on average (i.e., mean).

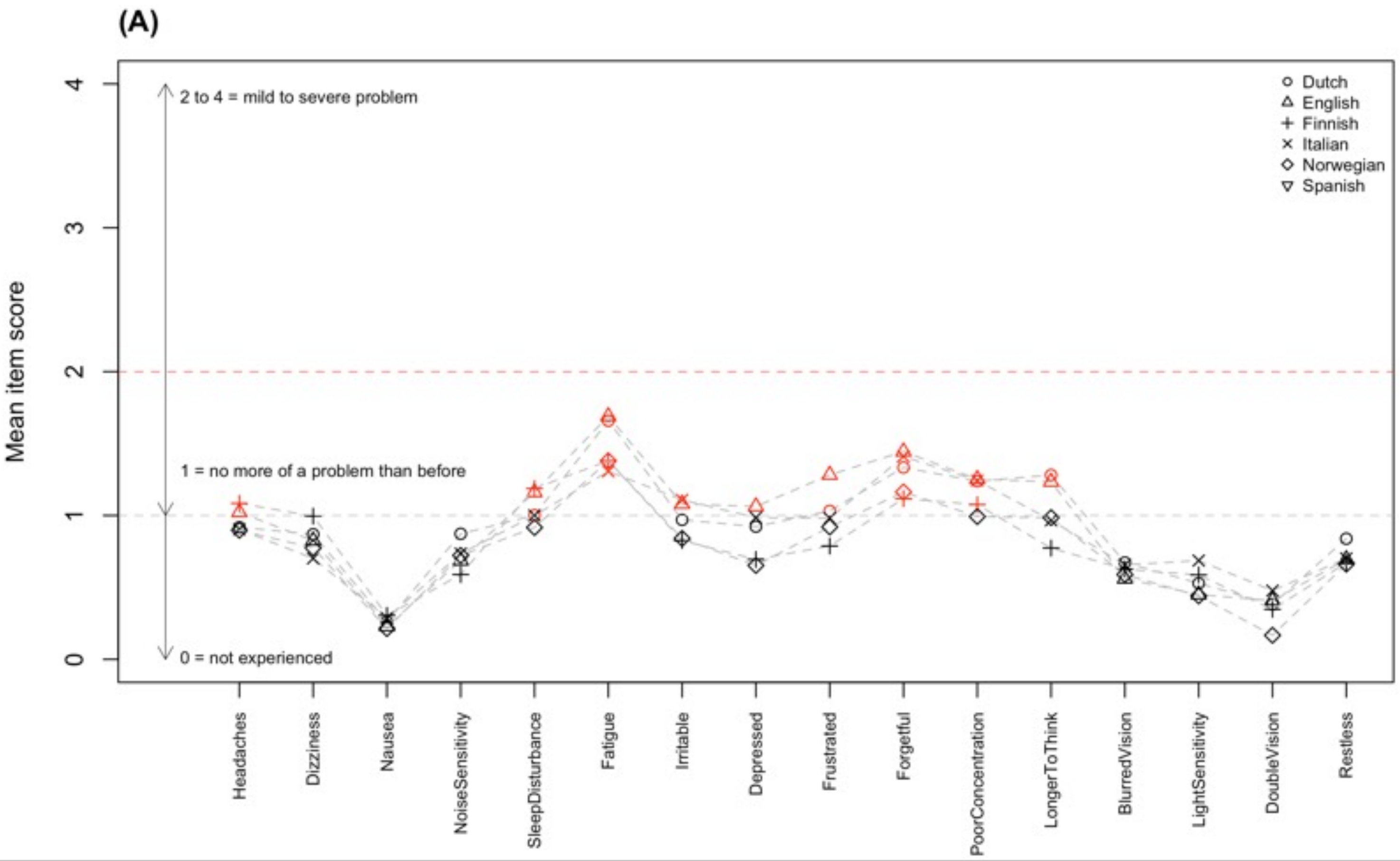

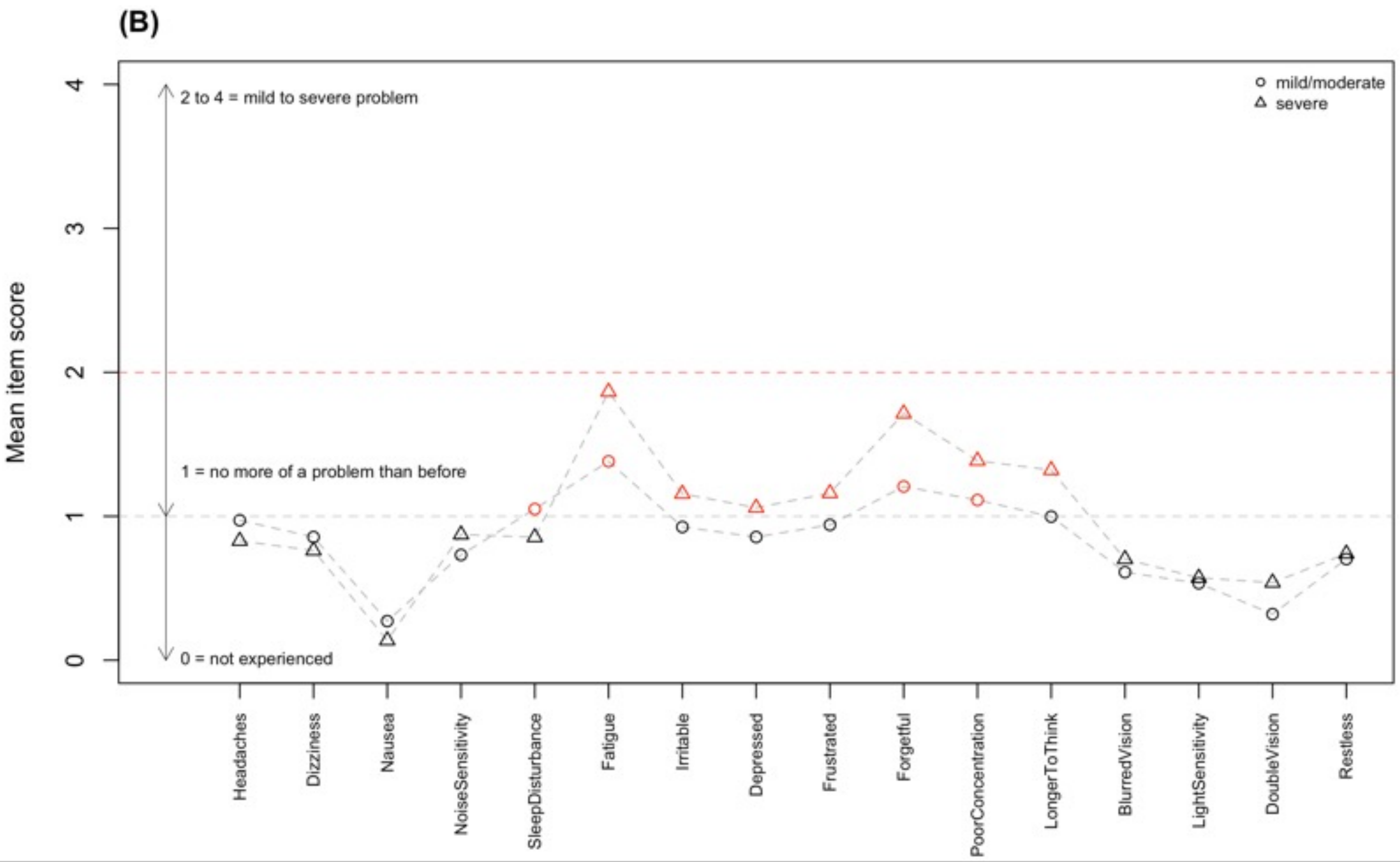

Supplement: Supplementary file 2 — Additional file 2. Supplementary figures S1. [file 41687_2023_632_MOESM2_ESM.pdf]
