# Supplementary material for: Factorial validity and comparability of the six translations of the Rivermead Post-Concussion Symptoms Questionnaire translations: results from the CENTER-TBI study
Source: J Patient Rep Outcomes. 2023 Sep 8;7:90. doi: 10.1186/s41687-023-00632-5 (PMC10491569; doi:10.1186/s41687-023-00632-5)

Fig. S2

Final model including somatic (soma), emotional (emo), and cognitive (cog) factors for the total study sample when treating “1” responses as “0”. The numbers depict standardized coefficients.

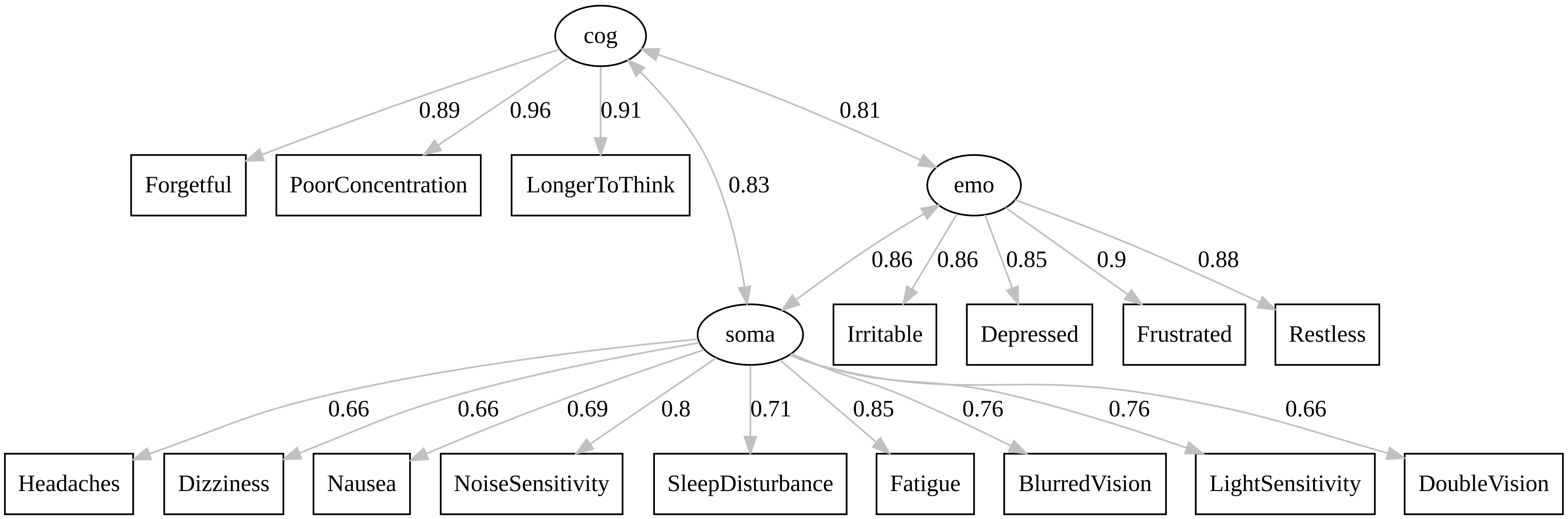

Supplement: Supplementary file 3 — Additional file 3. Supplementary figures S2. [file 41687_2023_632_MOESM3_ESM.pdf]
